# ReliableGenome: annotation of genomic regions with high/low variant calling concordance

**DOI:** 10.1093/bioinformatics/btw587

**Published:** 2016-09-07

**Authors:** Niko Popitsch, WGS500 Consortium, Anna Schuh, Jenny C Taylor

**Affiliations:** 1 Wellcome Trust Centre of Human Genetics, University of Oxford, Oxford, UK; 2 National Institute for Health Research (NIHR) Oxford Biomedical Research Centre, The Churchill Hospital, Old Road, UK; 3 Department of Oncology, University of Oxford, Oxford, UK

## Abstract

**Motivation:**

The increasing adoption of clinical whole-genome resequencing (WGS) demands for highly accurate and reproducible variant calling (VC) methods. The observed discordance between state-of-the-art VC pipelines, however, indicates that the current practice still suffers from non-negligible numbers of false positive and negative SNV and INDEL calls that were shown to be enriched among discordant calls but also in genomic regions with low sequence complexity.

**Results:**

Here, we describe our method ReliableGenome (RG) for partitioning genomes into high and low concordance regions with respect to a set of surveyed VC pipelines. Our method combines call sets derived by multiple pipelines from arbitrary numbers of datasets and interpolates expected concordance for genomic regions without data. By applying RG to 219 deep human WGS datasets, we demonstrate that VC concordance depends predominantly on genomic context rather than the actual sequencing data which manifests in high recurrence of regions that can/cannot be reliably genotyped by a single method. This enables the application of pre-computed regions to other data created with comparable sequencing technology and software. RG outperforms comparable efforts in predicting VC concordance and false positive calls in low-concordance regions which underlines its usefulness for variant filtering, annotation and prioritization. RG allows focusing resource-intensive algorithms (e.g. consensus calling methods) on the smaller, discordant share of the genome (20–30%) which might result in increased overall accuracy at reasonable costs. Our method and analysis of discordant calls may further be useful for development, benchmarking and optimization of VC algorithms and for the relative comparison of call sets between different studies/pipelines.

**Availability and Implementation:**

RG was implemented in Java, source code and binaries are freely available for non-commercial use at https://github.com/popitsch/wtchg-rg/.

**Supplementary information:**

Supplementary data are available at *Bioinformatics* online.

## 1 Introduction

Whole-genome resequencing (WGS) allows researchers to address a broad range of clinical and research questions at comparably low costs and with short turnaround times. For these reasons it is quickly becoming a central tool for genomic medicine and the accurate calling of small variants (SNVs and small INDELs) from WGS data plays a central role in most analyses. The currently observed amount of discordant variant calls between different state-of-the-art variant calling (VC) pipelines, however, indicates that the current practice still suffers from non-negligible numbers of false positives and negatives (Li, 2014; Motoike *et al.*, 2014; O’Rawe *et al.*, 2013; Ratan *et al.*, 2013, 2015).

It was shown that false positives and negatives are strongly enriched among calls that are discordant between different VC pipelines as these are often a consequence of imperfect data interpretation by the applied bioinformatics pipelines (Li, 2014; Motoike *et al.*, 2014; O’Rawe *et al.*, 2013). Consequently, one proposed practice to improve overall VC accuracy is to apply multiple VC pipelines to the same sequencing data and combine the results in order to reach a consensus from multiple algorithms (Cantarel *et al.*, 2014; Gézsi *et al.*, 2015). While this strategy may significantly increase VC accuracy it also greatly increases analysis costs and turnaround times which may be unfeasible in many real world situations. Nevertheless, such a consensus approach was used for the development of first genome-wide benchmarks that enable us to determine VC accuracy and reproducibility and thus pave the way for systematically improving these measures (Goldfeder *et al.*, 2016; Highnam *et al.*, 2015; Zook *et al.*, 2014). Another proposed filtering approach is to remove variants in particular genomic regions, e.g. regions with low sequence complexity. These regions were shown to harbour many false positive calls, mainly due to potential PCR and realignment errors. Simply removing all variant calls in such difficult regions is straightforward and did not compromise sensitivity significantly in the author’s evaluation (Li, 2014).

The method presented in this paper tries to augment these ongoing efforts by analysing patterns of discordance between call sets derived from the same sequencing data using different VC pipelines. One specific question that motivated this research was whether low call concordance is predominantly a property of the actual sequencing data quality or of the genomic location/context of the call in question. The latter option would become manifest in recurrent regions of discordance which would consequently allow us to predict such regions for future datasets, at least if those were created by comparable technology. Knowing genomic regions with high VC concordance *a priori* saves the effort of analysing every WGS dataset with multiple pipelines. Instead, one may either consider only the (reliable) calls in concordant regions for downstream analysis or alternatively concentrate additional efforts (e.g. multiple variant callers) solely on the small discordant fraction of the genome, thereby significantly reducing analysis and validation costs and times. Additionally, comparisons between datasets/studies would become easier and more reproducible if restricted only to variants in concordant regions. Above all, however, it would allow us to study recurrent regions of discordance in greater detail with the ultimate goal of uncovering the underlying causes for VC artefacts therein (cf. Li, 2014).

## 2 System and methods

Our method for calculating concordant and discordant regions in the genome consists of two main stages (cf. Supplementary Fig. S1). First, call sets for individual samples that were created with different VC pipelines are joined into single VCF files by iterating over all genomic positions with at least one non-filtered call in the input call sets. Let $C_{i,j}$ be the call sets for i∈1,…,N samples that were derived using j∈1,…,M different variant calling pipelines. The pipeline-specific call sets are joined into *N* single VCF files by iterating over all genomic positions with at least one non-filtered call in any of the *M* input files.

**Fig. 1.**
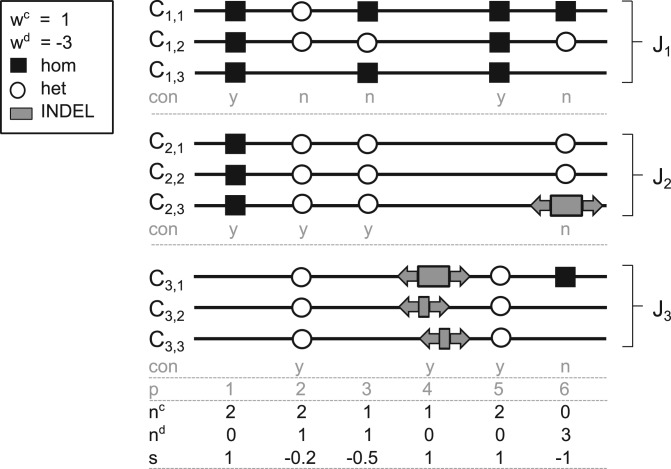
Concordance scoring example. The figure shows three call set groups (grouped horizontal black lines) derived from three different VC pipelines. The called genotypes of the individual calls are represented as follows: black rectangles: homozygous variant calls; white circles: heterozygous variant calls; shaded rectangles: INDELs. The letters *y* and *n* below each group indicate genotypes concordance or discordance among the VC pipelines; *p* is the index of the polymorphic positions; *n^c^* and *n^d^* are counts of concordant and discordant decisions respectively; *s* is the calculated concordance score using the weights $w^{c}=1$ and $w^{d}=-3$. Note that position 4 shows a concordant call because the extended INDEL intervals (indicated by arrows) overlap. INDEL calls are also compared based on their called genotype

For each such position we compare all called genotypes (in the VCF GT field) and write a merged variant call to a new joined VCF file *J_i_*. A merged call is written as being ‘discordant’ (by using a respective entry in the VCF FILTER field) if the called genotypes did not match or if some call sets $C_{i,j}$ did not contain a variant at this position. Otherwise it is written as being ‘concordant’ (using a ‘PASS’ filter). As INDELs are sometimes not represented in the same way or at the exact same genomic position by different VC pipelines (e.g. due to different variant alignment strategies), we decided to compare INDEL calls not simply by their coordinates but rather considered them matched if their associated 5′–3′ genomic intervals that were extended by *l* bases up-and downstream overlap between the different call sets, cf. Figure 1. We set *l* = 5 in this study.

The joined call sets *J_i_* are the input to the second stage of our algorithm that calculates a concordance score *s_p_* for each polymorphic position *p* in the input call sets $C_{i,j}$:

$s_{p}\in [-1,1]=\frac{n_{p}^{c}\cdot w^{c}+n_{p}^{d}\cdot w^{d}}{n_{p}^{c}\cdot w^{c}+|n_{p}^{d}\cdot w^{d}|}$ where $n_{p}^{c}$ and $n_{p}^{d}$ are the numbers of concordant respectively discordant calls in all *J_i_* at this genomic position *p* and $w^{c}>0$ and $w^{d}<0$ are configurable scoring weights, cf. Figure 1. A straightforward interpretation of this scoring schema is that negative scores correspond to ‘discordant’ positions, positive scores correspond to ‘concordant’ positions and scores close to zero correspond to a ‘neutral state’ where a decision could not be made due to contradictory or missing input data.

In a subsequent step, the concordance scores for all other genomic position (i.e. all positions for which there is no call in any $C_{i,j}$) is calculated by interpolation. For this, we consider genomic windows of size 2·x+1, centred on each position *p_k_* from the ordered set of all polymorphic positions in $C_{i,j}$ (we chose *x* = 1000 in this study). If the upstream polymorphic position $p_{k-1}$ is contained in this window then we linearly interpolate the score signal between $p_{k-1}$ and *p_k_*, otherwise we interpolate between $p_{k}-x$ and *p_k_* with the score $s_{p_{k}-x}:=0$ set to zero (the ‘neutral state’). The same is done for the downstream half of the window. An intuitive interpretation of this interpolation step is that each actual data point ‘radiates’ its score to its genomic neighbourhood with a strength that decreases linearly with distance. The final result of our algorithm is a genome wide concordance score signal as show in Supplementary Figure S2. From this signal we simply derive concordant and discordant genomic regions using configurable score thresholds *t_c_* and *t_d_* respectively. A more detailed description of the individual algorithmic steps and their implementation is given in the Supplement. We evaluated our approach by calculating a set of concordant regions from *N* = 219 WGS datasets that were subjected to *M* = 3 different VC pipelines using samtools (SAMT, Li *et al.*, 2009), GATK haplotype caller (GATK, DePristo *et al.*, 2011) and platypus (PLAT, Rimmer *et al.*, 2014). Details about the datasets and pipelines used are given in the Supplement. We tried several scoring schemas (data not shown) and finally picked one that minimizes the number of false-positives by setting *w_c_* = 1 and $w_{d}=-3\cdot w_{c}=-3$ (see discussion below). As we wanted to evaluate our tool as a binary classifier, we set *t_c _*=*_ _t_d_* which resulted in a genomic partition (RG) of concordant and discordant regions that covers the whole genome.

## 3 Results

We conducted three different evaluation experiments for measuring the performance of our method.

### 3.1 First experiment

**Fig. 2.**
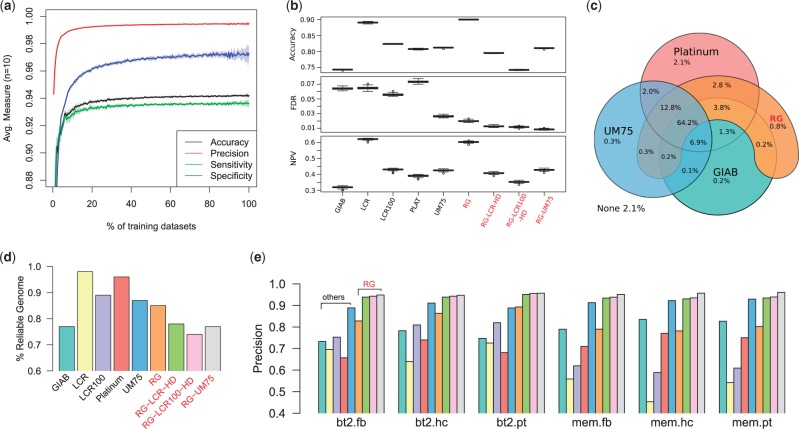
Statistics and evaluation results. (**a**) Results for evaluation experiment 1. The performance metrics shown were calculated by splitting the chr20 call sets from the described 219 WGS datasets into two random subsets and using one for training RG and the other as ground truth. The x-axis plots the percentage size of the training set. For each training set size we repeated the experiment 10X to avoid random artefacts. The plotted solid line corresponds to the mean value of the respective measure, the light-coloured corridor depicts the standard deviation. (**b**) Results for evaluation experiment 2. Accuracy (Acc=(TP+TN)/(TP+TN+FP+FN)), false discovery rate (FDR=FP/(FP+TP)) and negative prediction value (NPV=TN/(TN+FN)) boxplots for the various partition sets were calculated by using 34 independent WGS samples as ground truth. A complete set of performance metrics is given in Supplementary Fig S4. (**c**) Venn diagram showing the overlaps between four genomic partitions. The numbers show percentages of covered genomic positions that are considered reliable. 2.1% of the human reference genome (excluding assembly gaps) is not covered by any of the sets. (**d**) Bar plot showing the percentage of the human genome considered to be reliable/concordant per partition. (**e**) Results for evaluation experiment 3. The bar plot shows the precision for classifying heterozygous CHM1 calls as false positives. All call set labels are explained in the main text. Here, the combined *RG *+* UM75* partition as well as other *RG* derived set show low and constant false-positive rates of around 5-7%, by this outperforming other methods such as GIAB or PLAT about 3-4X

In the first experiment, we conducted i=1,…,215 evaluation runs and for each selected a random subset of size i from our WGS cohort. These subsets were used as training data whereas the remaining datasets were interpreted as ground truth. For each dataset, we considered the chr20 portion of the three pipeline-specific call sets and used our method to calculate partitions of concordant regions that were then evaluated as binary classifiers against the ground truth. We calculated accuracy, sensitivity and specificity of the resulting partition and plot the results in Figure 2a. The whole experiment was repeated 10X to avoid random artefacts. The main goal of this first evaluation experiment was to check the robustness of the RG algorithm. As expected, the calculated partitions become more accurate with increasing numbers of training datasets. The saturation of the curves (after about 10%, i.e. about 22 datasets in our cohort) can be explained by the rapidly decreasing number of additional data points that are added once all common polymorphic positions were covered. Eventually, our binary classifier reaches high values for precision (>99%) and specificity (>97%) while showing slightly worse sensitivity (>93%) which can partially be explained by the scoring schema used. Most importantly, the observed accuracy profiles can only be explained with high recurrence of concordant/discordant genomic regions meaning that concordance is predominantly determined by genomic position and not the actual read data/qualities in our cohort.

### 3.2 Second experiment

In the second evaluation experiment, we tried to measure how robustly we can apply a pre-calculated genomic partition to a different cohort and compared the results to similar approaches. For this, we first calculated a genome-wide genomic partition from all 219 WGS datasets mentioned above (*RG*) and then calculated an alternative genomic partition from 34 independent WGS datasets that were used as ground truth set in this experiment. These 34 datasets were mapped with a different read mapper but variants were called using the same VC pipelines as for the *RG* set (see Supplement for more details). We then measured how well we could predict concordant/discordant variant calls in these data using our genomic partition *RG* as well as four comparable sets of genomic regions that were proposed for filtering variants, namely the ‘reliable regions’ from the Genome-In-A-Bottle project (*GIAB*; Zook *et al.*, 2014), the ‘confident regions’ from Illumina’s Platinum project (*PLAT*; http://www.illumina.com/platinumgenomes/), a set of low-complexity regions that was recently suggested for variant filtering (*LCR*; Li, 2014) and a set of (yet unpublished) genomic regions from the same author (*UM75*; Heng Li, personal communication). We also derived a set *LCR100* by extending each low complexity region in *LCR* by 100bp upstream and downstream and a set *RG-UM75* that combines the two respective partitions. We furthermore created two derived genomic partitions (*RG-LCR-HD* and *RG-LCR100-HD*) from our *RG* set by excluding all *LCR* or *LCR100* regions and all genomic windows that showed high density (*HD*) of discordant calls. For this, we iterated over all variant calls in our 219 WGS datasets and counted the number of (stretches of) discordant calls that were interrupted by (stretches of) concordant calls within a genomic window that started 1000 bp upstream and ended 1000 bp downstream of the respective call. We then created a genomic partition by merging all such genomic windows that contained at least 10 such counts (see Supplement for further details). The respective genomic coverages of all used genomic partitions are depicted in Figure 2d.

We plotted accuracy, false discovery rate (FDR) and negative prediction value (NPV) in Figure 2b. The Figure shows that our method achieves the highest accuracy of all compared sets while showing the lowest FDR which is what we optimized for with the chosen scoring schema. Removing *LCR* and *HD* regions or regions from the *UM75* set from our partition may further lower the FDR, however, at the costs of reduced accuracy. Our method shows the second-highest NPV, only slightly behind LCR, demonstrating that it also harbours less false negatives (i.e. true concordant calls that were classified as being discordant) than most other approaches (cf. Supplementary Fig. S4).

### 3.3 Third experiment

In our final evaluation experiment, we measured the performance of our genomic partition for predicting potential false positives/sequence artefacts. Following along the lines of Li (2014) we used variant calls derived from a WGS dataset of the CHM1hTERT (CHM1) cell line for this evaluation. As CHM1 is assumed to be haploid we considered all heterozygous variant calls as false positives. We downloaded the call sets from this publication that were prepared using two different mappers (bowtie2 [bt2] and bwa mem [mem]) and three different variant callers (FreeBayes [fb], GATK HaplotypeCaller [hc] and Platypus [pt]), extracted all heterozygous calls with a quality >30 and measured the precision of different genomic partitions to classify them as false positives (Fig. 2e). The *RG*-derived partitions clearly outperform all other partitions in this evaluation, reaching >95% precision when *LCR*, *HD* and/or *UM75* regions are subtracted.

### 3.4 Supplementary analysis of discordant positions

Finally, we comprehensively analysed all variant positions that we found in the 219 WGS datasets by calculating various statistics and present the results in the Supplement. Table 1 contains a summary of our findings and provides references to the respective supplemental Figures that show the data. In particular, our sequence context analysis (Supplementary Section 6.1) confirms that discordant calls are located predominantly in regions with low sequence complexity and that their context composition differs significantly from concordant variants which could be utilized for the further characterization of problematic DNA regions and the improvement of variant filtering strategies. Note that our results are in good concordance with recently published research on sequence characteristics and polymorphism rates in the human genome, cf. Aggarwala and Voight (2016); Sahakyan and Balasubramanian (2016).

**Table 1.** This table summarizes the results of several statistics for discordant calls derived from over 34 million polymorphic genomic positions in the described 219 WGS datasets

	Discordant variant positions…	SNV	INDEL	Figure
Variant calling and cohort related	Are less abundant in the cohort	Yes	No	S7
	Show less classification agreement (more intermediate scores)	Yes	Yes	S10
	Have less contributing datasets in the cohort	No	No	S11
	Have lower variant qualities	Yes	Yes	S17
	Potentially violate Hardy-Weinberg equilibrium more often	Yes	n/a	S19
	Are more often multi-allelic	Yes	n/a	S22a
Genomic location related	Are more abundant in low-mappability regions	Yes	Yes	S14
	Are less covered	Yes	Yes	S15
	Are closer to adjacent INDELs	Yes	Yes	S16
	Are closer to INDEL locations in the cohort	Yes	Yes	S16
	Are less abundant in genes/exons	Yes	Yes	S20
External DB related	Are less abundant in dbSNP	Yes	Yes	S8
	Have allele frequencies that correlate worse with population AFs	Yes	yes	S12
	Are considered less deleterious by CADD	Yes	n/a	S18
Sequence context related	Are enriched in annotated LCR	Yes	Yes	S20
	Show reduced Ts/Tv ratios	Yes	n/a	S22ff
	Are enriched in AT rich regions	Partially	Partially	S23ff
	Show reduced sequence context complexity	Yes	Yes	S23ff

A more detailed discussion of these data is given in the Supplement.

## 4 Discussion

Although germline variant calling is considered a solved problem by many, researchers still observe low overlap between different state-of-the-art VC pipelines and tools. This discordance between different methods not only hinders data comparison, integration and reproducibility; it also indicates non-negligible numbers of false positives and negatives that are introduced due to differing interpretation of sequencing data by current VC pipelines. This stands in contrast to the increasing demands for highly accurate and reproducible VC sets from WGS experiments, in particular in clinical environments (Koboldt *et al.*, 2010; Taylor *et al.*, 2015; The Genome of the Netherlands Consortium, 2014). With WGS now rapidly entering the clinic, VC discordance could become a serious concern for the emerging field of precision medicine and related areas.

Our evaluation of deep WGS data provides strong evidence that VC concordance for such datasets depends predominantly on genomic context rather than on quality properties of the actual sequencing data. This enables the *a priori* calculation of genomic partitions with high variant calling concordance that can then readily be applied to additional data analysed by the same pipeline. Our method differs from comparable previous efforts in that it incorporates data from many different WGS datasets, thereby capturing more of the data’s variance. It is applicable to arbitrary variant sets (note, however, that the current version of RG supports only diploid genomes) and can thus be used to calculate high-concordance regions in a large range of scenarios. Our evaluation confirmed that our classifier improves with more training data which means that we can expect even higher accuracies when applying RG to larger cohorts such as the recently sequenced set of 10,000 human genomes (Telenti *et al.*, 2016) or the emerging *100,000 Genomes* dataset. On the other hand, we reached high performance measures after only about 20 datasets which makes RG also applicable to small and medium sized cohorts.

The genomic partition created by our method naturally depends to some extent on the used technology to create the sequencing data, the employed VC pipelines, the configured parameters (mainly scoring schema and interpolation window size) and also on the data itself. However, as shown in this study, the influence of the actual sequencing data is much smaller than we initially expected. Also, although not thoroughly evaluated in this study, we found evidence that our genomic partition is quite robust with respect to slightly differing VC pipelines. Experiments 2 and 3 demonstrate that a partition that was derived from a different cohort can be used to accurately predict regions of discordance/unreliable VC in data that were created using different read mapping and variant calling algorithms. This may render RG a useful tool for the comparison and integration of data from different studies that was analysed using various bioinformatic pipelines or even sequencing technologies. Besides applying the same RG partition on data from multiple cohorts, one could also easily intersect RG partitions calculated from the individual cohort data to calculate shared regions of concordance.

Genomic partitions such as the one presented in this study allow us to focus on the ‘hard to call’ fraction of the genome where current VC algorithms are often in disagreement and where false positives and negatives accumulate. This enables the concentration of available resources on the small part of the genome that cannot be confidently genotyped by a single method, e.g. by applying computationally intensive consensus calling algorithms that do not scale to the whole genome (cf., Cantarel *et al.*, 2014; Gézsi *et al.*, 2015; Goode *et al.*, 2013). Alternatively, users may decide to simply ignore calls in such error-prone regions. Filtering WGS call sets with our genomic partition is straightforward and can effectively reduce false positive calls without risking many false negatives. This might be particularly interesting in clinical scenarios as it reduces validation costs and shortens turnaround times. The relevance of this is underlined by our supplementary data analysis that shows, in accordance with recently published results by (Goldfeder *et al.*, 2016), that a considerable fraction of discordant calls are found in genomic regions of high clinical interest (genes and exons). Furthermore, our analysis provides additional evidence for the potential pollution of public annotation databases (such as population allele frequency databases, cf. Fig. S9) with false positives.

Most importantly, however, such genomic partitions may help in identifying the underlying causes for false positives and negatives and consequently improve the VC accuracy of existing tools. Variant call sets are currently filtered mainly by per-variant quality values that are calculated from a mix of sequence dependent (e.g. per-base quality values) and location-dependent (e.g. read mapping qualities) features. The supplemental analysis of our genomic partition shows that quality values work well yet are not perfect for separating concordant and discordant calls. As we and others showed that false positives are strongly enriched among discordant calls, we conclude that current variant quality values are also limited in filtering those. Augmenting the location-dependent features that are already considered by current quality value calculations, we identified/confirmed several cohort-wide properties (such as distance to cohort-wide INDEL locations, transition/transversion ratios, or AT- enrichment in the sequence context) that seem to differ substantially between concordant and discordant variant calls. These features show considerable variance among datasets and neither feature alone seems sufficient for the accurate prediction of concordant of discordant calls. Our method captures this variance by integrating the decisions from multiple algorithms across multiple datasets, and in this regard it is not surprising that it substantially outperforms previous works that were derived from single datasets (GIAB reliable regions, Illumina Platinum regions) or single sequence-dependent features (LCR).

Our findings are consistent with previously reported causes for artefacts such as PCR errors, alignment errors in repetitive regions and around INDELs but also the incompleteness of the used reference genome. Actually we expect the fraction of discordant regions to decrease when using the latest version of the human reference genome which would reduce the number of artefacts stemming from copy number variations (CNV) or missing paraloguous sequences, cf. Pickrell *et al.* (2011); Li (2014); Miga *et al.* (2015). Particularly, regions with low sequence complexity were reported to be major sources of variant calling errors and our analysis largely confirms this yet shows that call discordance is not restricted to such regions. By combining our partition with LCR annotations and regions of high discordance density we were able to reach high and constant precision for filtering false positive calls, outperforming GIAB, PLAT and LCR 3-4X.

Unannotated copy number variations (CNVs) are another likely source of discordant variants. The used UM75 partition was created by integrating regions of low read mapability and/or low sequence complexity with regions that are enriched with ‘aberrant’ variant calls. The latter were computed by clustering regions with negative inbreeding coefficients in 1000 Genomes Project phase III data (see Supplement for details). By doing this, UM75 excludes regions that are likely mis-assembled in the human reference genome or are enriched with common CNVs in the 1000G data. Such regions are likely to harbour many false-positives in the experiment 3 data (but also the other evaluation data) and UM75 thus complements our genomic partition well as demonstrated by the good results when combining both sets.

## 5 Conclusion and perspectives

In this work we presented a first straight-forward method for calculating genomic partitions that are likely to harbour concordant/discordant variant calls in WGS resequencing experiments when applying different VC pipelines. Our method can be applied to arbitrary VC pipelines and the resulting genomic partitions can be used for variant filtering, annotation and prioritization or for focusing computational resources on hard-to-analyse regions of the genome. It may further be useful for the development, benchmarking and optimization of VC algorithms and pipelines with the goal of improving their accuracy and for the relative comparison of VC results across different studies and pipelines. A limitation of the current study is that it was conducted only with deep WGS data from a single sequencing technology and it is yet unclear whether the results are reproducible with other kinds of sequencing data.

Possible future work includes the investigation of this matter, the comparison of somatic variant callers (that show even less concordance in current studies, cf. Alioto *et al.*, 2015) and the improved explicit integration of identified features with high classification value into our method. We furthermore plan to further analyse the sequence context around discordant variants and integrate our genomic partitions with other data, such as structural variation data and recombination hotspot annotations.

## Supplementary Material

Supplementary Data
