# Supplementary material for: ReliableGenome: annotation of genomic regions with high/low variant calling concordance
Source: Bioinformatics. 2016 Sep 7;33(2):155–60. doi: 10.1093/bioinformatics/btw587 (PMC5903559; doi:10.1093/bioinformatics/btw587)
Supplement: Supplementary Data [file btw587_supp.pdf]

ReliableGenome : Annotation of Genomic Regions with High/Low Variant Calling Concordance
Supplementary Materials

Niko Popitsch, WGS500 Consortium, Anna Schuh and Jenny C. Taylor

Contents

1 Datasets **2**
1.1 VCF files . . . 2
1.2 Variant calling pipelines . . . 3
1.2.1 Platypus . . . 3
1.2.2 GATK HaplotypeCaller . . . 3
1.2.3 Samtools . . . 3
1.3 Other genomic partitions . . . 3

2 Ethics statement **3**

3 RG Algorithm **4**
3.1 RG JOIN procedure . . . 4
3.1.1 RG JOIN commandline . . . 4
3.2 RG CALC procedure . . . 5
3.2.1 Scoring schema . . . 5
3.2.2 Union file generation . . . 5
3.2.3 Score interpolation . . . 5
3.2.4 Extraction of concordant and discordant genomic regions . . . 5
3.2.5 RG CALC commandline . . . 7
3.2.6 RG high density region calculation . . . 7

4 Evaluation **7**
4.1 Experiment 1 - Internal Evaluation . . . 7
4.2 Experiment 2 - Evaluation against other WGS datasets . . . 7
4.3 Experiment 3 - Evaluation with a haploid cell line . . . 7

5 Implementation **7**

6 Characterization of discordant positions **10**
6.1 Sequence Context Analysis . . . 20

7 Authors' contributions **30**
7.1 WGS500 Consortium . . . 30

8 Supplemental References **31**

1 Datasets

1.1 VCF files

1. The core dataset of this study consists of VCF files derived from deep whole genome sequencing (WGS) alignments of the WGS500 project [17]. These 30X WGS alignments were previously created by mapping deduplicated, paired 100bp Illumina HiSeq reads to GRCh37d5 (hs37d5, the hg19-derived human reference genome that was used in the 1000 Genomes Project) using bwa+stampy [8, 11]. We created VCF files from these alignments using the variant calling (VC) pipelines described below. More detailed information (e.g., detailed coverage statistics) about the WGS500 data sets and the bioinformatics pipelines that were used for their creation can be found in the Supplementary information of [17].

   Our final set of concordant and discordant regions was calculated from VCFs derived from 219 of these WGS500 datasets and is accessible at `https://github.com/popitsch/wtchg-rg`. We also calculated a partition of concordant genomic regions from the publicly available subset of these data consisting of 9 genomes accessible under the ENA accession number ERP010230. This partition and a chr20 subset of all used input VCF files are also accessible at the above-mentioned URL. The complete VCF files are available on request from the author.

2. For evaluating the prediction of concordant/discordant regions in different datasets we used 34 independently sequenced germline WGS data sets from a currently ongoing pilot study for the 100,000 Genomes Project (in preparation) for which we created VCF files using the same VC pipelines as for our main dataset (see below). The respective 40X alignments were created by mapping deduplicated 125bp paired Illumina reads to GRCh37 with isaac [14]. Figure S1 plots the coverage of these data sets.

3. For measuring the precision in the CHM1hTERT (CHM1) cell line data, we downloaded call sets created with FreeBayes, GATK HaplotypeCaller and Platypus from `http://bit.ly/1g8XqRt` as described in [6].

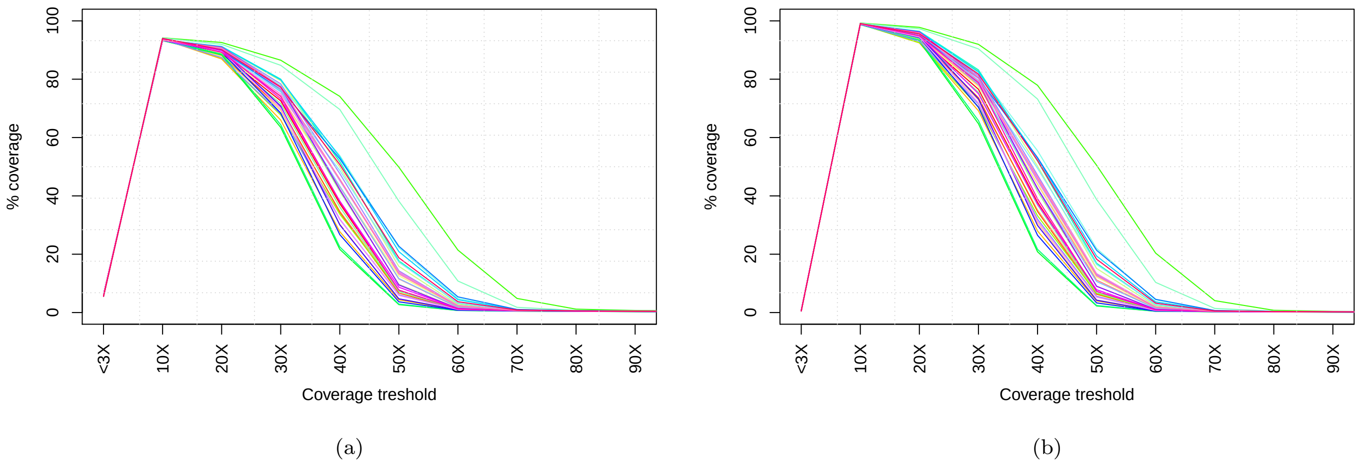

Figure S1: Read coverage across the whole genome (a) and in genes (b) in the 34 validation datasets used for experiment 2 of the main paper.

1.2 Variant calling pipelines

We used the following commandlines to call variants with Platypus, GATK's HaplotypeCaller and samtools. Please note that variants were called from individual BAM files and not in "joint" mode, i.e., the genotyping algorithms did not have access to cohort allele frequency information.

1.2.1 Platypus

We used the following commandlines to call variants with Platypus v0.7.9.3 [15] with default parameters and split complex variants (e.g., MNPs) with vcflib [4].

```
python Platypus.py callVariants --refFile=<hs37d5> --bamFiles=<BAM> \
     --logFileName=<LOG> -o <RAW.vcf>
vcflib vcfallelicprimitives <RAW.vcf> > <FINAL.vcf>
```

1.2.2 GATK HaplotypeCaller

The following commandlines were used to call variants with GATK's HaplotypeCaller v3.2-2 [3].

```
gatk -nct 4 -T HaplotypeCaller -rf BadCigar -R <hs37d5> --dbsnp <DBSNP-141> \
     -I <BAM> -o <RAW.vcf>
gatk -T SelectVariants -R <hs37d5> -V <RAW.vcf> -selectType SNP \
     -o <RAW.SNP.vcf>
gatk -T SelectVariants -R <hs37d5> -V <RAW.vcf> -selectType INDEL -o \
     <RAW.INDEL.vcf>
gatk -T VariantFiltration -R <hs37d5> -V <RAW.SNP.vcf> \
     --filterExpression "QD < 2.0 || FS > 60.0 || MQ < 40.0 ||
       MQRankSum < -12.5 || ReadPosRankSum < -8.0" \
     --filterName GatkRecommendedHardFilter -o <FILTERED.SNP.vcf>
gatk -T VariantFiltration -R <hs37d5> -V <RAW.INDEL.vcf> \
     --filterExpression "QD < 2.0 || FS > 200.0 || ReadPosRankSum < -20.0" \
     --filterName GatkRecommendedHardFilter -o <FILTERED.INDEL.vcf>
gatk -R <hs37d5> -T CombineVariants --variant:snps <FILTERED.SNP.vcf> \
     --variant:indels <FILTERED.INDEL.vcf> -o <FINAL.vcf> \
     -genotypeMergeOptions PRIORITIZE -priority snps,indels
```

1.2.3 Samtools

We called and filtered variants with samtools v1.1 [9] and vcfutils.pl using the following commandlines:

```
samtools mpileup -ugf <hs37d5> <BAM> | bcftools call -vmO z -o <RAW.vcf>
cat <RAW.vcf> | vcfutils.pl varFilter | bgzip -c > <FINAL.vcf.gz>
```

1.3 Other genomic partitions

- GIAB reliable regions were downloaded from `ftp://ftp-trace.ncbi.nih.gov/giab/ftp/`.
- Illumina Platinum Confident Regions were downloaded from `http://www.illumina.com/platinumgenomes/`.
- LowComplexity regions were downloaded from the link described in [6].
- UM75 regions were given to us by Heng Li (personal communication). Briefly, UM75 is the union of the following genomic partitions:
  - Regions of low mapability that were defined as regions where less than half of the 75-mers overlapping a particular genomic partition could be mapped uniquely with bwa (allowing at most one mismatch or gap).
  - Regions of low sequence complexity as determined by mDUST (`ftp://occams.dfci.harvard.edu/pub/bio/tgi/software/seqclean/`).
  - Regions enriched with aberrant SNP calls. Here, SNP calls from the 1000 Genomes Project with negative inbreeding coefficient that violate the Hardy-Weinberg equilibrium were clustered and the resulting clusters were extended by 150bp upstream and downstream.

2 Ethics statement

The core datasets used in this paper come from the WGS500 Project, a collaboration between the University of Oxford, Oxford Biomedical Research Centre and Illumina, Inc, to sequence 500 genomes of clinical relevance. Samples were accepted from patients where positive findings would have immediate clinical translational relevance in terms of clinical diagnosis, prognosis, genetics counselling and reproductive options, or treatment selection. The WGS500 umbrella project consists of many smaller sub-projects, focusing on particular diseases. All patients in this study gave

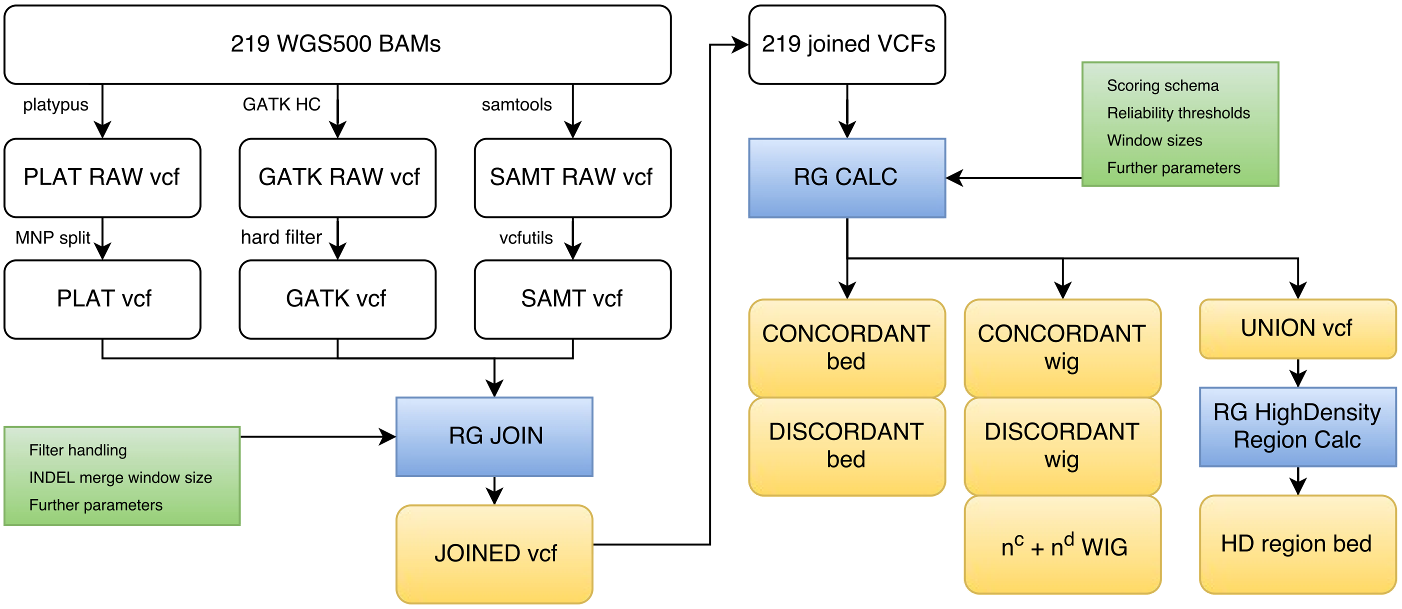

Figure S2: Overview and main result files of the RG method. White boxes: intermediate results; blue boxes: algorithmic core steps; green boxes: configuration parameters; orange boxes: result files.

written informed consent. The relevant research ethics committee (REC) reference numbers are: Central Oxfordshire Research Ethics Committee (05/Q1605/88), Hammersmith and Queen Charlottes and Chelsea REC (06/Q0406/151), NRES Committee South Central Oxford B (12/SC/0381), NRES Committee South Central Southampton A (12/SC/0044), NRES Committee North WestHaydock (03/0/97 version 3), NRES Committee Yorkshire & The HumberSouth Yorkshire (10/H1310/73), Oxfordshire Research Ethics Committee (06/Q1605/3), Oxfordshire Research Ethics Committee B (04.OXB.017; 09/H0605/3) and Oxfordshire Research Ethics Committee C (09/H0606/74; 09/H0606/5), Riverside Research Ethics Committee (09/H0706/20) and Southampton and South-West Hampshire REC A (06/Q1702/99). The research conformed to the Helsinki Declaration and to local legislation.

3 RG Algorithm

Figure S2 depicts an overview of our algorithm. First, we applied the above-mentioned variant calling pipelines to the WGS500 BAM files, leaving us with 3 x 219 filtered and normalized variant call sets (VCF files). Then we estimated concordance in these files and merged them (Section 3.1) which resulted in 219 joined VCF files. Finally, these files were used to calculate a genomic partition of concordant and discordant regions as described in Section 3.2.

3.1 RG JOIN procedure

Variant calls from the three different pipelines were combined (joined) by iterating over all genomic positions with at least one non-filtered call in one of the call sets. For such positions, we compared all called genotypes (in the VCF GT field) and wrote a merged variant call to the joined VCF file with a filter string "discordant" if the genotypes did not match, or "PASS" otherwise. As INDELs are sometimes not represented in the same way or at the exact same genomic position by variant callers (e.g., due to differing "left alignment" strategies), we decided to compare INDEL calls not simply on their leftmost coordinate and alternate allele entry but rather considered them matched if their associated 5'-3' genomic intervals that were extended by l bases up- and downstream overlapped between the different call sets. We set $l = 5$ in this study (commandline parameter $indelMergeWin$).

3.1.1 RG JOIN commandline

The following commandline was used for joining the VCF files. The resulting VCF files were sorted and indexed and then further annotated.

```
java -Xmx12g -jar bin/wtchg-rg-1.0.jar join \
  -d <GATK.VCF> -dl GATK \
  -d <PLAT.VCF> -dl PLAT \
  -d <SAMT.VCF> -dl SAMT \
  -o <SNV.out.vcf> \
  -oi <INDEL.out.vcf> \
  -dontCheckSort -dropAllFiltered -indelMergeWin 5
```

3.2 RG CALC procedure

3.2.1 Scoring schema

The 219 joined VCF files were then altogether used to calculate concordant and discordant genomic regions. For this, we iterated over all genomic positions with at least one (concordant or discordant) call in the set of joined VCFs. For each such position p we then calculated a concordance score s_p using the following formula:

$$s_p \in [-1, 1] = \frac{n_p^c \cdot w^c + n_p^d \cdot w^d}{n_p^c \cdot w^c + |n_p^d \cdot w^d|}$$

where n_p^c and n_p^d are the numbers of concordant respectively discordant calls in all J_i at this genomic position p and $w^c > 0$ and $w^d < 0$ are configurable scoring weights.

A straightforward interpretation of this scoring schema is that genomic positions with negative scores correspond to "discordant" genomic positions due to the observed discordant situations in the used training data. Accordingly, more positive scores correspond to more "concordant" positions and a score of zero corresponds to a "neutral" state. We chose $w^c = 1$ and $w^d = -3$ as a weighting schema based on some initial experiments (data not shown). The rationale behind this rather conservative schema was that researchers might use our genomic partition to quickly get a concordant call set by applying one single state-of-the-art variant calling pipeline to the estimated concordant regions while putting more (computational) effort into creating a good call set for the estimated discordant regions of the genome. Accordingly, the chosen weighting schema strongly punishes discordant situations in the training data in order to minimize the overall number of false positives (cf. the low FDR in Figure 2b of the main manuscript). Other weighting schemas might be more appropriate in other scenarios and are easily configurable via commandline parameters.

3.2.2 Union file generation

After this step, RG creates a union VCF file containing one merged call for each considered position. RG then adds the calculated score s_p and other statistics such as allele frequencies and the number of datasets containing a call at this genomic position to VCF INFO fields. The resulting union VCF file can then directly be loaded into a genome browser for debugging purposes or used for further downstream analysis (e.g., for extracting genomic regions with high or low allele frequencies in a cohort). RG uses this file to calculate regions with a high density of discordant calls as described below.

3.2.3 Score interpolation

The union file contains scores for all variable positions in the considered training cohort but does not say anything about all the other genomic positions. Our detailed analysis of all variable positions, however, showed that the reliability of a considered genomic position is to a large part influenced by the reliability of its genomic surrounding (see Section 6). For this reason, RG implements a score interpolation model in which each call "radiates" its score to its genomic neighbourhood with a strength that decreases with distance (cf. Figure S4). Specifically, score interpolation is done by considering a genomic window of size $2 \cdot x + 1$, centred on each called position p. If there are other calls upstream of p within this window, then the score signal is linearly interpolated between p and the closest upstream call. If this is not the case, then the score signal is linearly interpolated between $p - x$ and p with the score $s_{p-x} := 0$ (the neutral state). Correspondingly, if there are other calls downstream of p within the genomic window, then the score signal is linearly interpolated between p and the closest downstream call and if not, it is interpolated between p and $p + w$ (again, with $s_{p+x} := 0$).

The genomic window size is configurable and we chose $x = 1000$ in this study (the influence of the window size on the performance of our algorithm is briefly discussed in Figure S3).

3.2.4 Extraction of concordant and discordant genomic regions

RG calculates a genome-wide score signal by interpolating the calculated scores between all considered positions as described above (Figure S4). This signal[1] is then used to extract concordant and discordant genomic regions by considering two score thresholds t_c and t_d: genomic regions with scores above t_c are written to a "concordant" BED file, genomic regions with scores below t_d to a "discordant" BED file. For this study, we selected a rather conservative threshold $t_c = +0.5$ for calculating concordant genomic regions. As we wanted to evaluate RG as a binary classfier, we chose $t_d = t_c$, i.e., all genomic regions that were not concordant were considered discordant. But we would like to point out that also different thresholds for t_c and t_d can be chosen which would then result in an additional set of genomic regions with scores between these thresholds. This might, e.g., be beneficial in a scenario where researchers want to treat regions with high discordance different from such "intermediate" regions (e.g., if they want to ignore calls in the discordant set and apply different analysis strategies/more validation to the intermediate set).

[1] Note that this score signal can be exported by RG as a WIG file (cf. `http://genome.ucsc.edu/goldenpath/help/wiggle.html`) for debugging or development purposes.

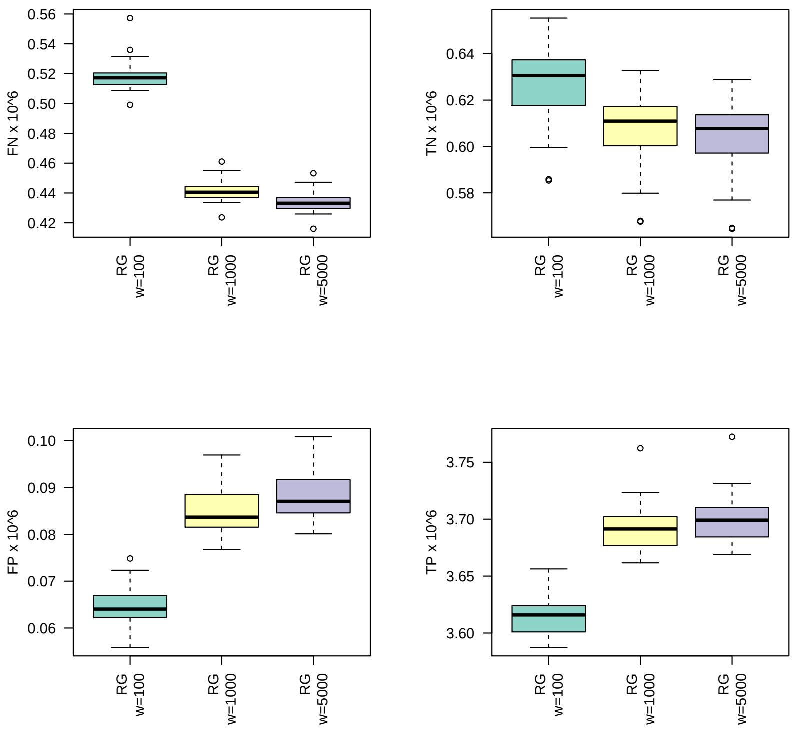

Figure S3: Influence of different interpolation window sizes (100, 1000, 5000) on the number of false negatives (FN), true negatives (TN), false positives (FP) and true positives (TP) when measured as in evaluation experiment 2 (cf. Figure S5). We configured RG with $w = 1000$ for the results presented in this paper as this window size showed a good tradeoff between these measures.

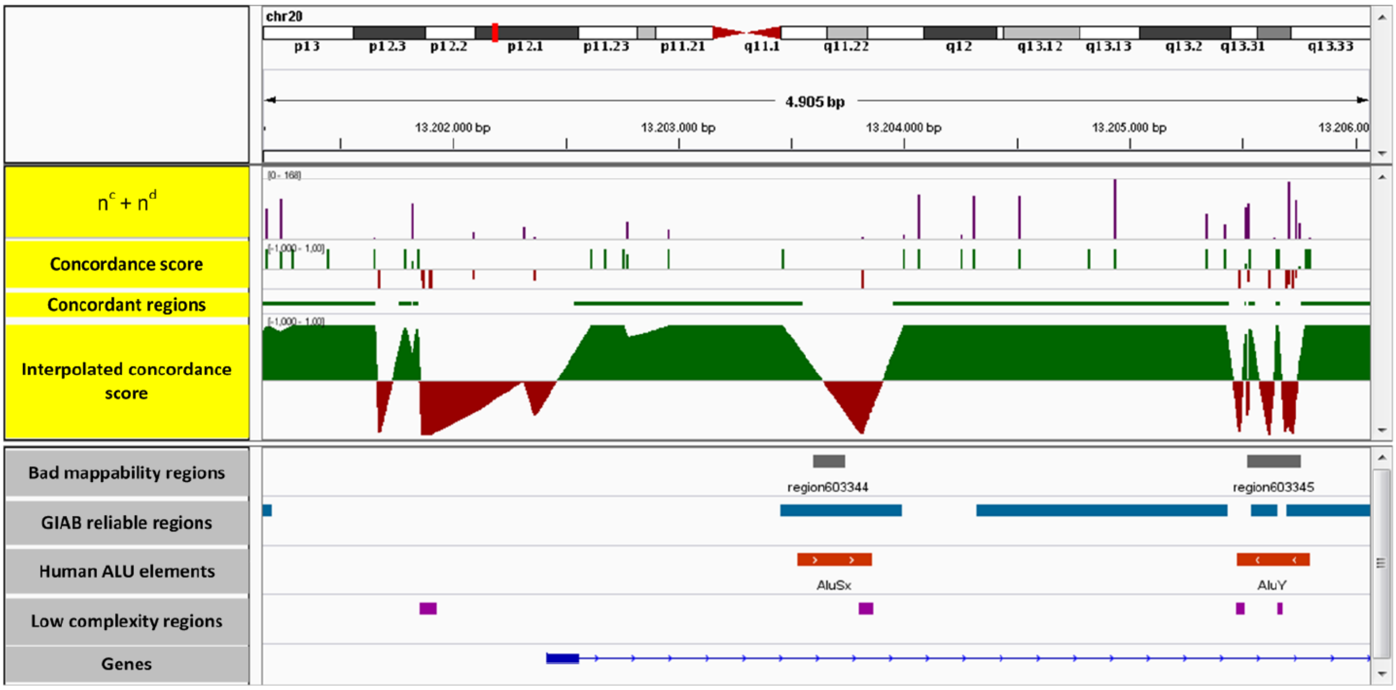

Figure S4: IGV screenshot showing the main RG result files (yellow tracks) and various genomic annotation tracks (grey tracks). The $n^c + n^d$ signal shows the number of training datasets that contained a call by at least one of the variant calling pipelines at the respective genomic position. The "Concordance score" signal shows the calculated score s_p at these genomic positions (colours: red for negative scores and green otherwise). The "Interpolated concordance score" track shows the described score interpolation between neighbouring calls or window endpoints respectively (none visible in this screenshot). The "Concordant regions" track shows the concordant genomic regions as calculated from the interpolated signal with a cutoff of $t_c = +0.5$. The annotation tracks show regions with low mappability as calculated by ARGOS [13], GIAB reliable genomic regions [19], Human ALU elements as downloaded from UCSC (http://genome.ucsc.edu/), low complexity regions [6] and RefSeq gene annotations.

3.2.5 RG CALC commandline

The following commandline was used for calculating concordant and discordant genomic regions from the joined VCF files.

```
java -Xmx12g -jar bin/wtchg-rg-1.0.jar calc \
  -o <OUTDIR> \
  -w 1000 \
  -scoringSchema 1,-3 \
  -thresholds 0.5,0.5 \
  -dontCheckSort -v
```

3.2.6 RG high density region calculation

To calculate regions with a high density of discordant calls (HD regions), we iterated over all calls in the created union file and counted the number of (stretches of) discordant calls that were interrupted by (stretches of) concordant calls within a genomic window that started 1000bp upstream and ended 1000bp downstream of the respective call. We then created a BED file by merging all such genomic windows that contained at least 10 such counts. We found 3,369,732 such windows in our data which resulted in 78,174 (merged) genomic HD regions.

4 Evaluation

In the following we describe the conducted evaluation experiments in more detail.

4.1 Experiment 1 - Internal Evaluation

In this experiment we considered only the calls from chromosome 20 of the described 219 WGS datasets and split them into a training and an evaluation subset as follows: In 215 consecutive runs, the set of 219 call sets was split into a *training* and an *evaluation* subset so that the training subset contained $n = 1, .., 215$ random call sets. These call sets were then used to calculate RG concordant and discordant regions using the described parameter settings and performance was measured by interpreting the remaining call sets as ground truth. For example, an discordant position in a truth-set would be counted as false positive (FP) if it was found in an RG-calculated concordant region, etc. The whole experiment was repeated 10 times and the averaged resulting performance measures were plotted in Figure 2a of the main manuscript.

RG-derived partitions become more accurate with increasing numbers of training datasets. The accuracy curve showing fast saturation after about 30 datasets at 94%. Eventually, the binary classifier reaches very high values of precision ($> 99\%$) and specificity ($> 97\%$).

4.2 Experiment 2 - Evaluation against other WGS datasets

In this evaluation experiment we trained RG with the described cohort of 219 WGS datasets and used call sets derived from an independent set of 34 deep WGS datasets as a ground truth set. We then measured how well the calculated genomic partitions can predict whether calls in the employed truth sets are concordant or discordant, thus once more interpreting genomic partitions as binary classifiers. Detailed performance metrics of this evaluation are plotted in Figures S5 and S6.

4.3 Experiment 3 - Evaluation with a haploid cell line

For this experiment, we downloaded VCF files from `ftp://ftp.1000genomes.ebi.ac.uk/vol1/ftp/technical/working/20140312_chm1_alignment_heng/vcfs/` and measured the precision of different genomic partitions to correctly classify heterozygous variant calls as false positives as described in the main manuscript.

5 Implementation

RG was implemented in Java, source code and binaries are available for non-commercial use at `https://github.com/popitsch/wtchg-rg`. The evaluation experiments were conducted on a server equipped with 2 Intel Xeon E5-2640 processors and a total of 256 GBytes of RAM. RG was run using Java 1.7.0 75 64Bit with 12 Gbytes of maximum heap size (-Xmx switch). The measured wall clock times for calculating an RG partition from 219 joined input VCF files on a single CPU thread was about 4 hours.

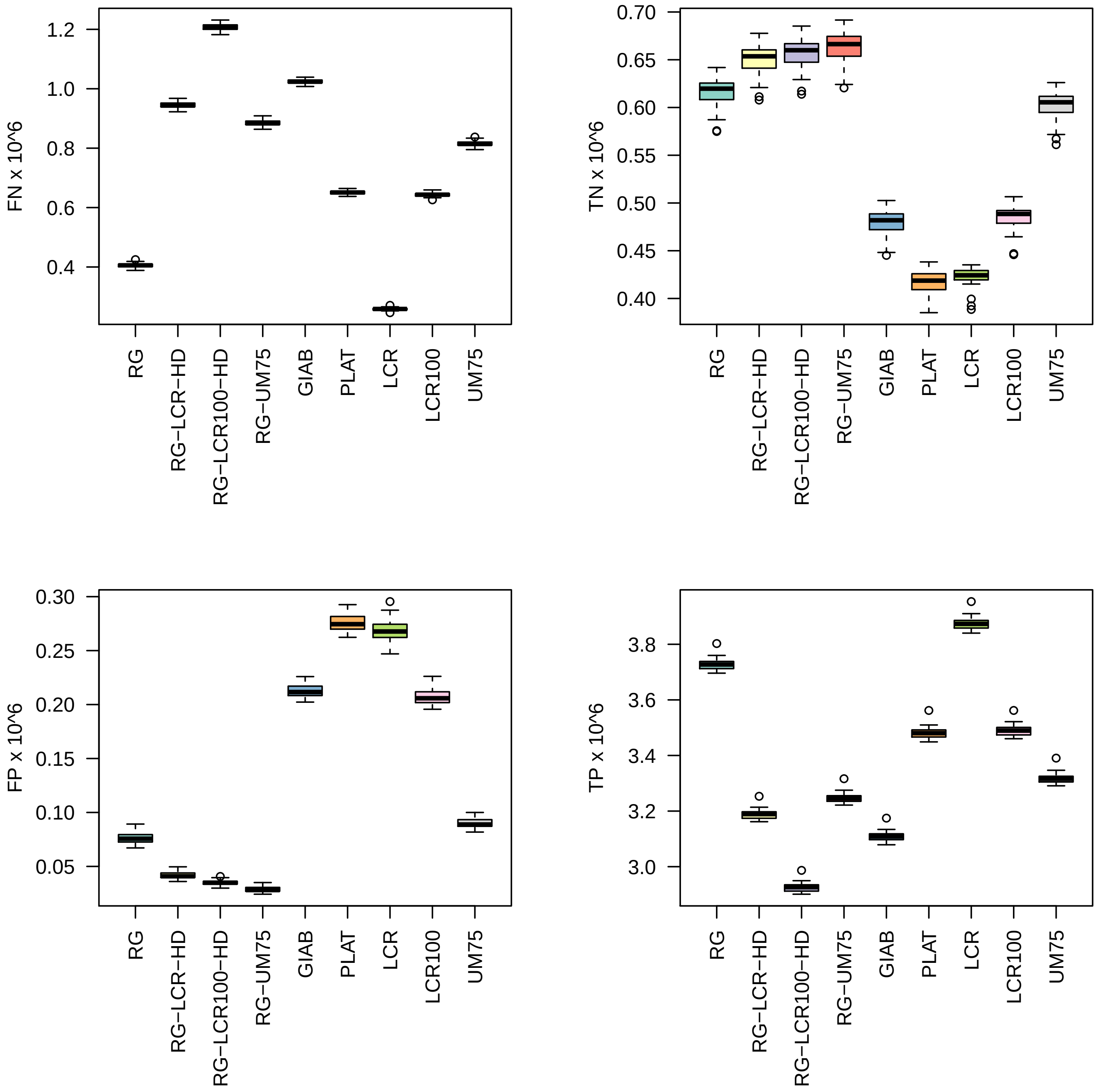

Figure S5: False negatives (FN), true negatives (TN), false positives (FP) and true positives (TP) for evaluation experiment 2. The RG-derived partitions clearly show the lowest numbers of false-positives (i.e., discordant positions that are classified as concordant) and the highest numbers of true negatives (i.e., discordant positions that are really discordant). Sets: RG: reliable genome partition from 219 WGS500 datasets; GIAB: Genome-In-A-Bottle reliable regions; PLAT: Illumina Platinum regions; LCR: Low-Complexity regions; HD: Regions with high densities of discordant calls; LCR100: LCR regions, extended by 100bp up/downstream; RG-LCR-HD: RG regions with LCR and HD regions removed; RG-LCR100-HD: RG regions with LCR100 and HD regions removed; RG-UM75: RG regions with UM75 regions removed.

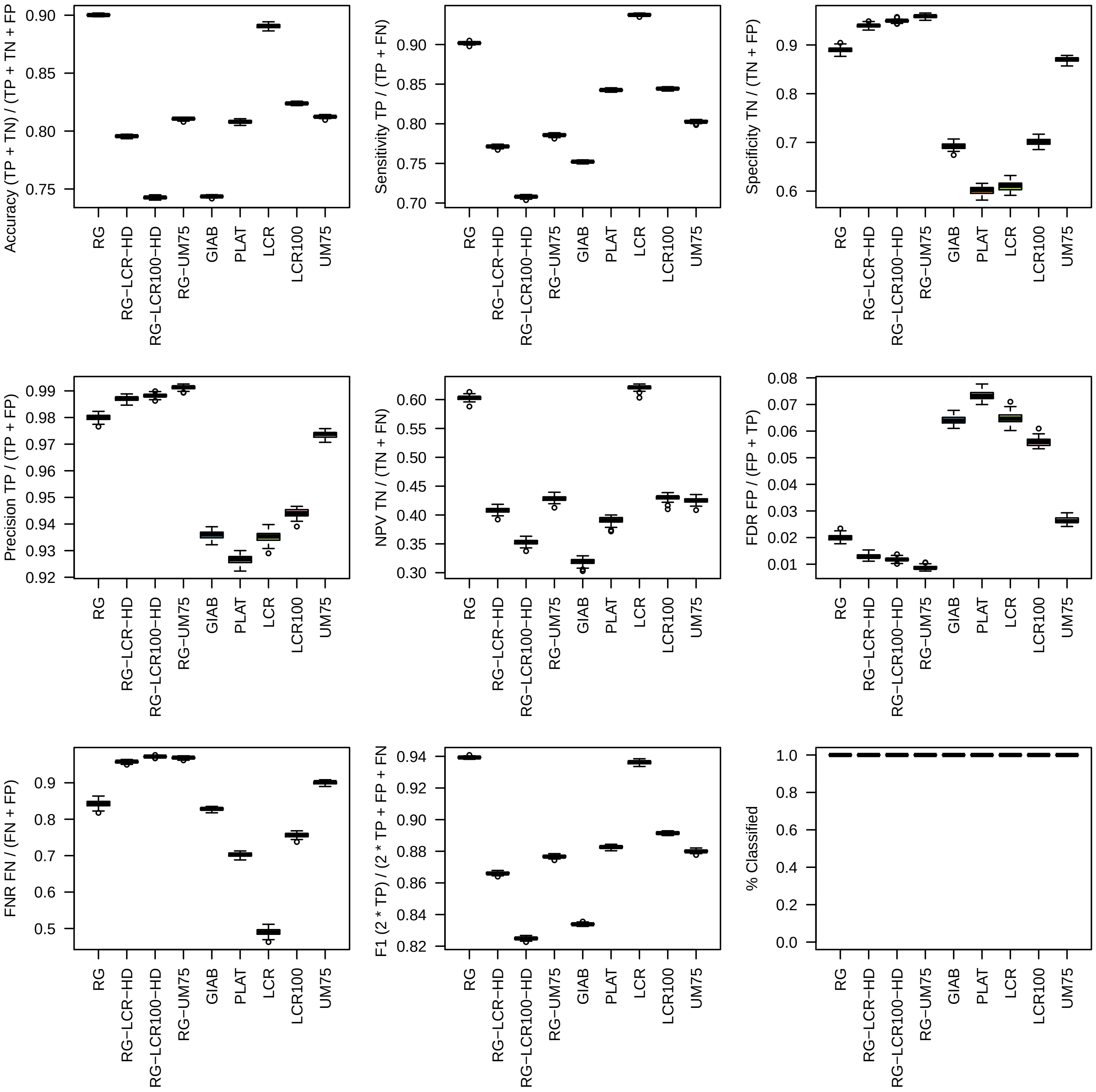

Figure S6: Various performance metrics calculated by measuring against 34 unrelated WGS datasets in evaluation experiment 2. The RG-derived partitions outperform the other partitions with respect to specificity and precision and show the lowest FDR. Sets as in Fig S5.

6 Characterization of discordant positions

In order to learn more about the underlying causes for discordant calls, we extracted several statistics from the alignments and call set files that were summarized in Table 1 in the main manuscript and plotted in the following. If not indicated otherwise, the following graphs were thus calculated by considering over 34 Mio positions that were called at least once in the described WGS500 dataset. The calls were then further subdivided into the following subsets (where appropriate):

- The set All contains all positions.
- The sets Con and Dis contain the subsets of positions that were considered as concordant or discordant by RG respectively.
- The sets $CCC, CCD, \ldots, CDC$ consider the context of a variant. The first letter describes the status (C: concordant, D: discordant) of the neighbouring upstream variant, the middle letter describes the status of the actually considered variant and the 3rd letter describes the status of the adjoining downstream variant. Figure S7 shows the fractions of these categories for SNVs and INDELs.

We calculated each statistic independently for SNVs and INDELs where appropriate and provide the following analyses:

- Fractions of concordant and discordant calls per category, Figure S7
- Occurrence in dbSNP, Figure S8
- Variant counts and densities per chromosome, Figure S9
- Concordance score density distributions, Figure S10
- Numbers of datasets contributing to the calls, Figure S11
- Correlations between measured allele frequencies (AF) in the cohort and expected (population) AF, Figure S12
- Variant caller contributions to concordant/discordant calls, Figure S13
- Mappability around variant calls, Figure S14
- Coverage around variant calls, Figure S15
- Distance to adjacent INDEL (locations), Figure S16
- Variant quality distributions, Figure S17
- CADD score distributions, Figure S18
- Hardy-Weinberg Equilibrium violations, Figure S19
- Fractions of concordant and discordant calls in genomic subregions (e.g., genes and exons), Figure S20
- Analysis of sequence context around variants, Section 6.1

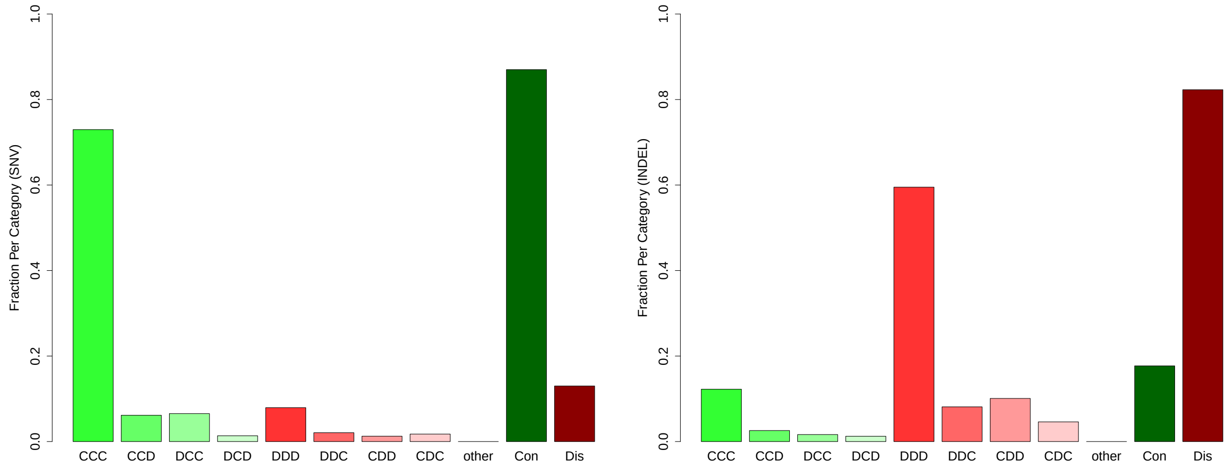

(a) (b)

Figure S7: Fractions of concordant and discordant calls per category for (a) SNVs and (b) INDELs. The majority of SNV calls was concordant while the majority of INDEL calls was considered discordant by RG.

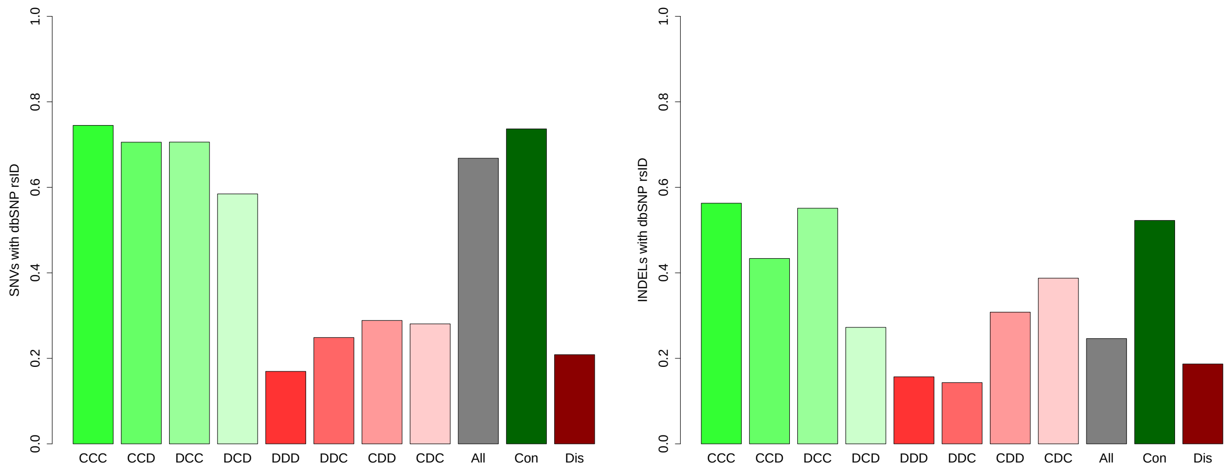

(a) (b)

Figure S8: SNV (a) and INDEL calls (b) with associated dbSNP IDs. About 3/4 of all concordant SNV and 1/2 of all concordant INDEL calls are recorded in dbSNP while discordant calls show a much reduced percentage (about 20%).

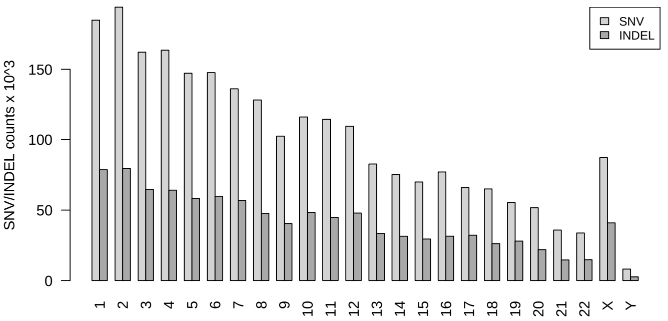

(a)

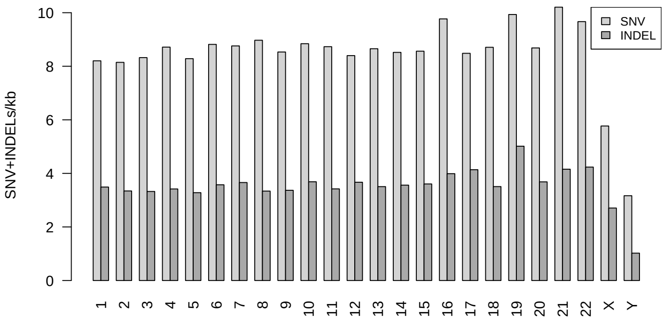

(b)

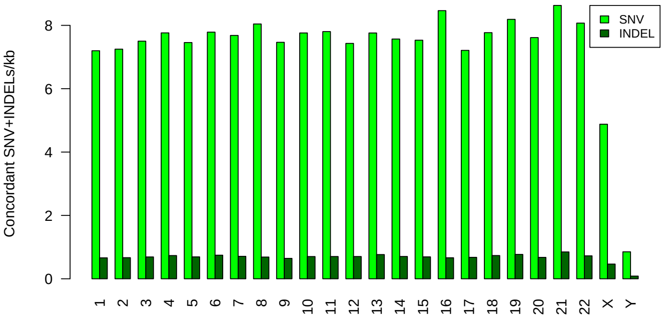

(c)

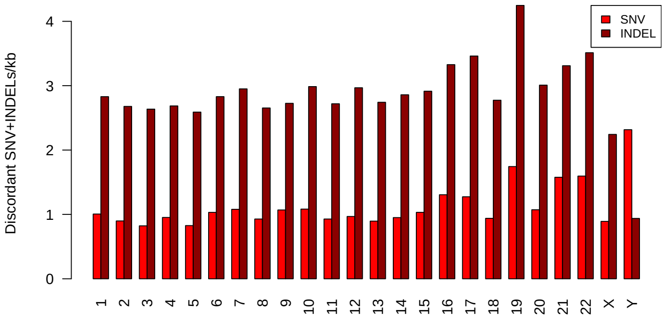

(d)

Figure S9: SNV and INDEL counts (a) and densities for all (b), concordant (c) and discordant (d) positions. Note that chromosome 19 (the most gene rich chromosome with the highest GC content) shows a slightly increased density of discordant SNVs.

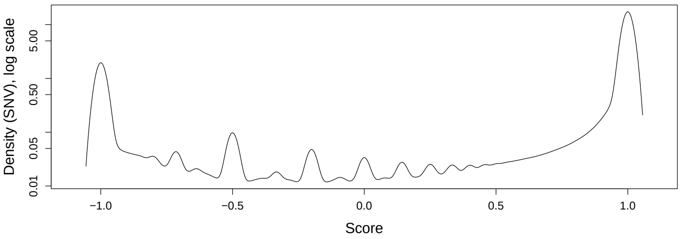

(a)

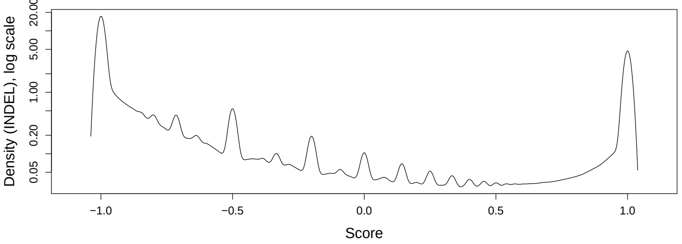

(b)

Figure S10: Score densities for (a) SNVs and (b) INDELs. The plots reveal various intermediate scores between -1 and $+0.5$ for discordant calls (the weighting schema was $+1/-3$, the score cutoff was 0.5) but no such scores for concordant calls (between $[+0.5, +1]$). Intermediate scores are a result of a mixture of concordant and discordant decisions for a particular position.

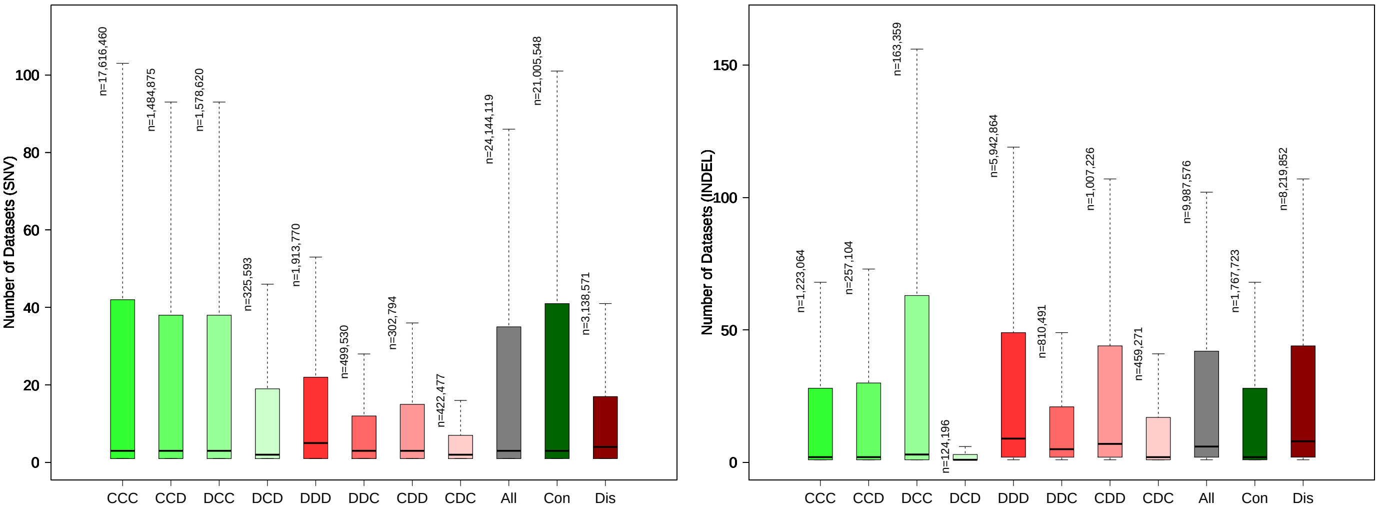

(a) (b)

Figure S11: Number of datasets contributing to a call (i.e. $n^c + n^d$ in Fig. 1 of the main manuscript) for (a) SNVs and (b) INDELs. The numbers of contributing calls per category are plotted next to the box whiskers. Discordant positions show a higher median for this measure for SNVs and INDELs but a smaller interquartile range for SNVs when compared to concordant calls.

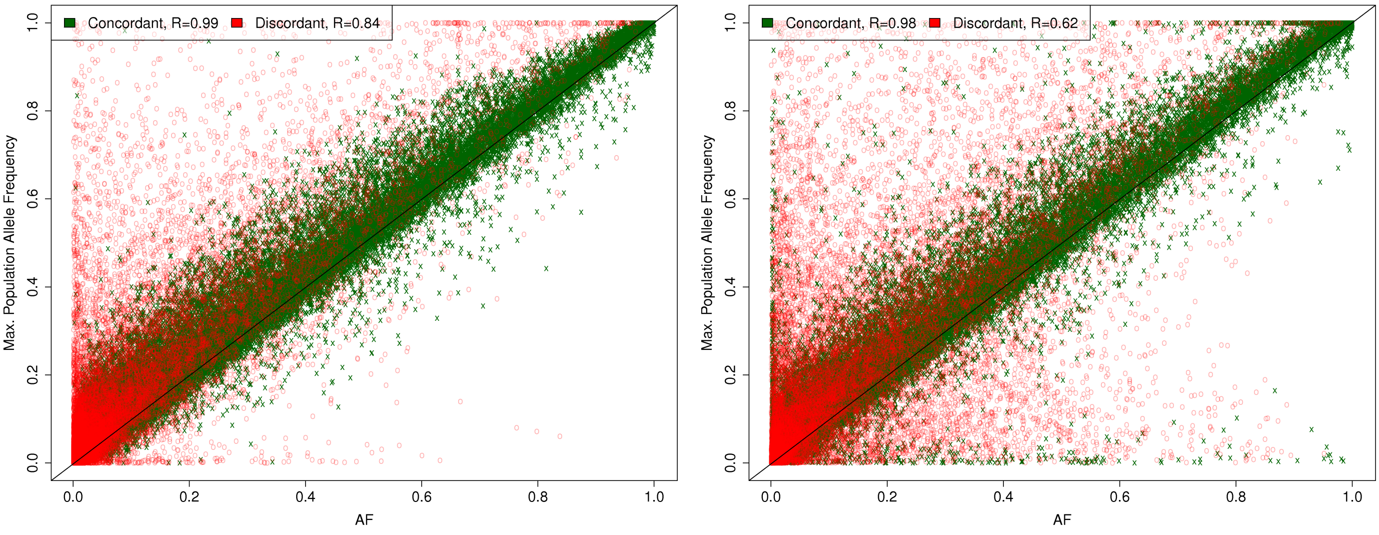

(a) (b)

Figure S12: Correlation between measured allele frequency in the cohort and expected (population) allele frequency for concordant (green) and discordant (red) SNVs (a) and INDELs (b). Only a random subset of the actual data points was plotted ($n = 100,000$), Pearson correlation coefficients were calculated from all data points. Population allele frequencies were calculated by taking the maximum value found in four different databases (ExAC, ESP6500, 1000G and UK10K) which might explain why the observed frequencies for concordant regions are slightly underestimated. Concordant positions show high correlation to the population genetics data, discordant positions show much reduced correlation.

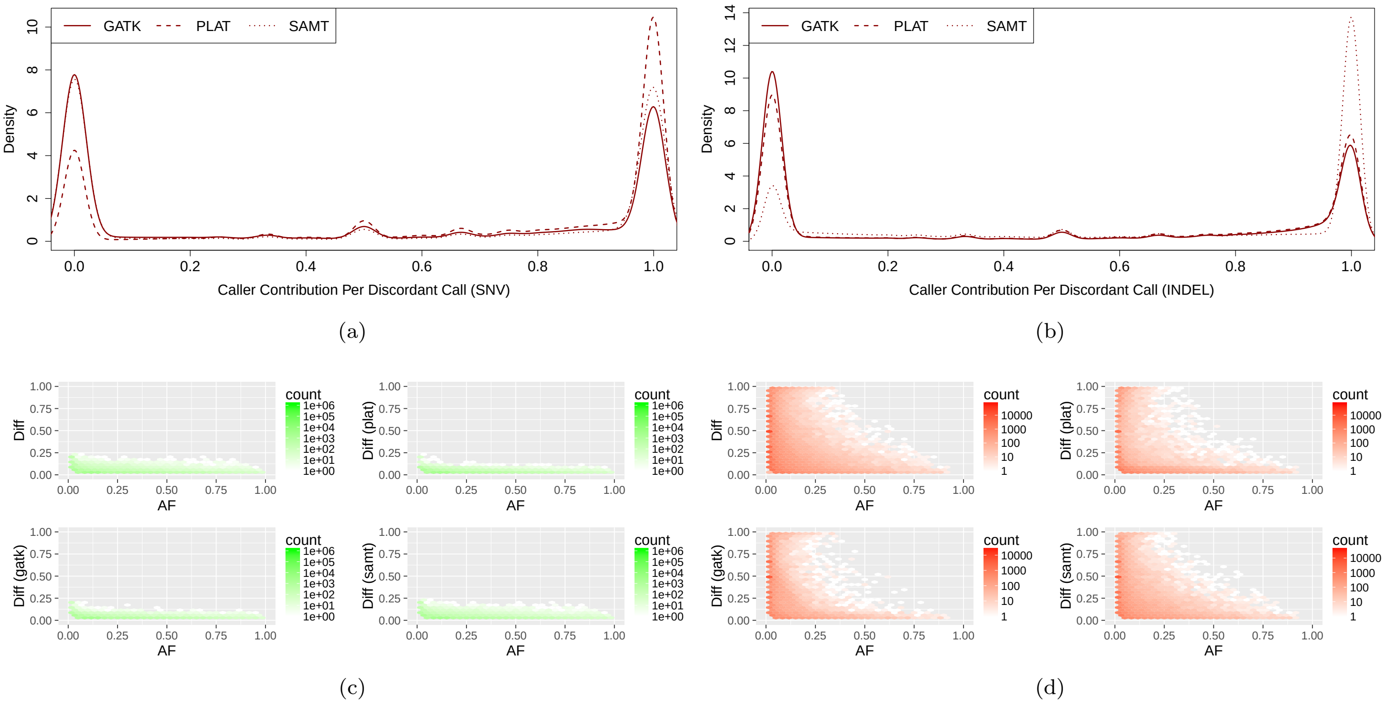

Figure S13: Distributions of the contributions of individual variant calling pipelines ("caller percentage", CP) for SNVs (a,c,d) and INDELs (b). The plotted CP_{caller} values for a given variant correspond to the number of variant caller-specific call sets containing a (heterozygous or homozygous) call divided by the number of total call sets containing the corresponding call (made by any variant caller). For example, if a variant was called (by any VC) in 20/219 datasets and it was called in 10/219 datasets by samtools, then the corresponding CP_{SAMT} value would be 0.5. Subfigures a+b show density plots of CP for discordant calls only. Subfigures c+d plot the difference between highest and second-highest CP values for a variant against the cohort allele frequency using the ggplot2/stat_binhex R package. A large "diff" value thus means that the corresponding variants were called predominantely by one caller but not the other two. The different plots show the results for concordant (c, green) and discordant (d, red) variants. The upper left plots contain all such data points, while the three caller-specific plots contain only the variants for which the respective CP value of the caller was above 0.9 (i.e., the caller contributed significantly to the call). Generally, we observe higher "diff" values for small cohort allele frequencies (AF) as these correspond to situations where discordance in few datasets could easily lead to high CP difference values. As expected we do not observe such large "diff" values for concordant calls across the full range of cohort AFs. For discordant calls we see large "diff" values for small cohort allele frequencies for all three variant callers. In a summary, the plots reveal that no single variant calling method is responsible for all discordant calls (independent of cohort allele frequency). They also shows that the used GATK pipeline contributed least to the sets of discordant calls and that the used samtools pipeline called a majority of the discordant INDEL calls. Please note, however, that the three employed variant callers differ in the way how they deal with suboptimal read alignments. While GATK's HaplotypeCaller and Platypus realign/assemble reads around variants which helps to avoid artefacts especially around INDEL regions, samtools does not conduct such a step which may explain its higher contribution to the discordant INDEL calls. However, as reported elsewhere such realignment steps are often also not optimal, especially in LCR regions and it will be interesting to see how pure assembly-based variant calling methods (e.g., [7]) deal with such situations.

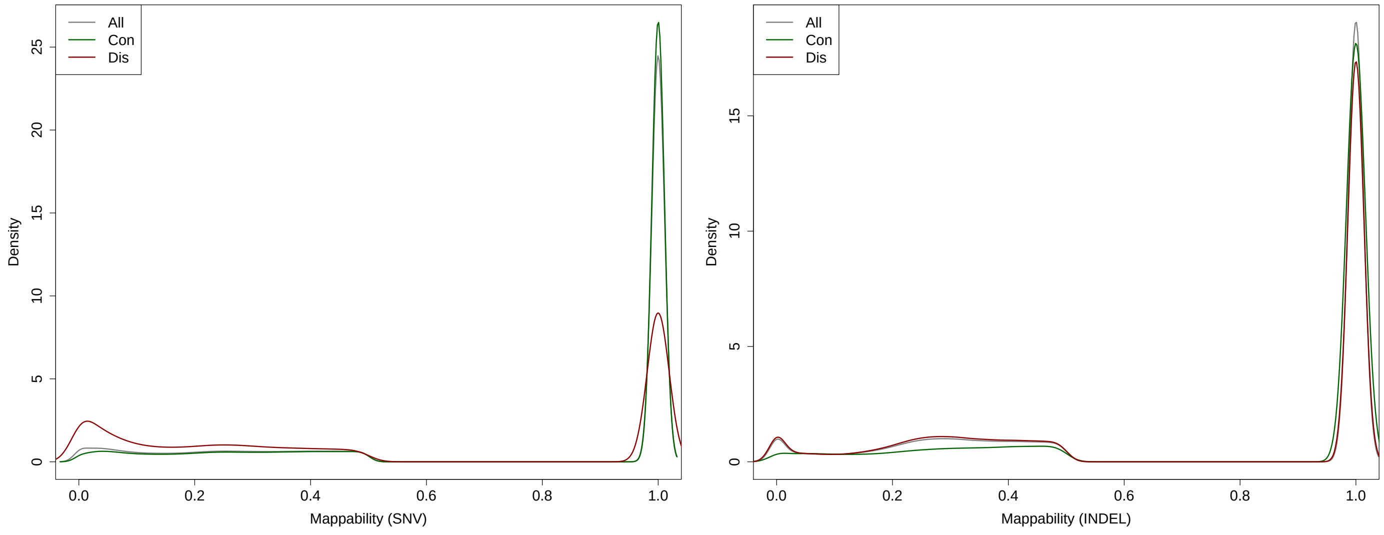

(a) (b)

Figure S14: Mappability around SNVs (a) and INDELs (b). We used ARGOS ISS scores ($rl = 200, step = 20$) for estimating the mappability of the respective genomic positions [13]. Low-mappability regions harboured more discordant positions.

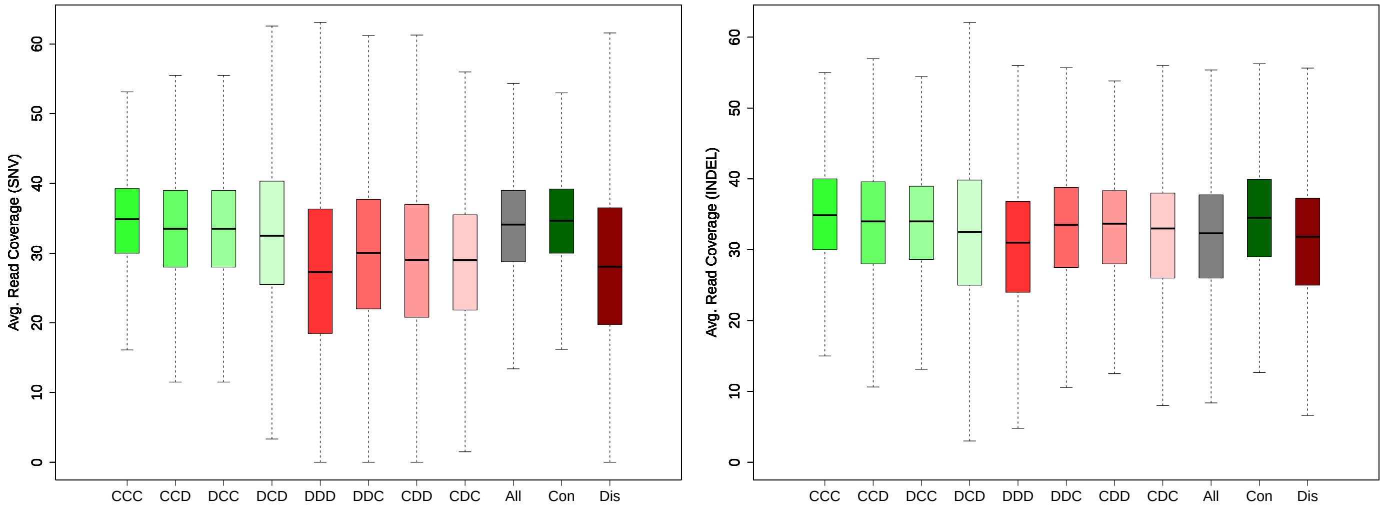

(a) (b)

Figure S15: Average read depth in genomic regions around SNVs (a) and INDELs (b). We calculated the average coverage in the 219 WGS alignments using CODOC [12]. Discordant positions show reduced read coverage which can to some extent be explained by the reduced mappability, see Figure S14. Neither mappability nor coverage, however, seem good classifiers for predicting concordance on their own in our deep WGS alignments. We speculate, however, that both properties would have a much larger impact in shallow datasets.

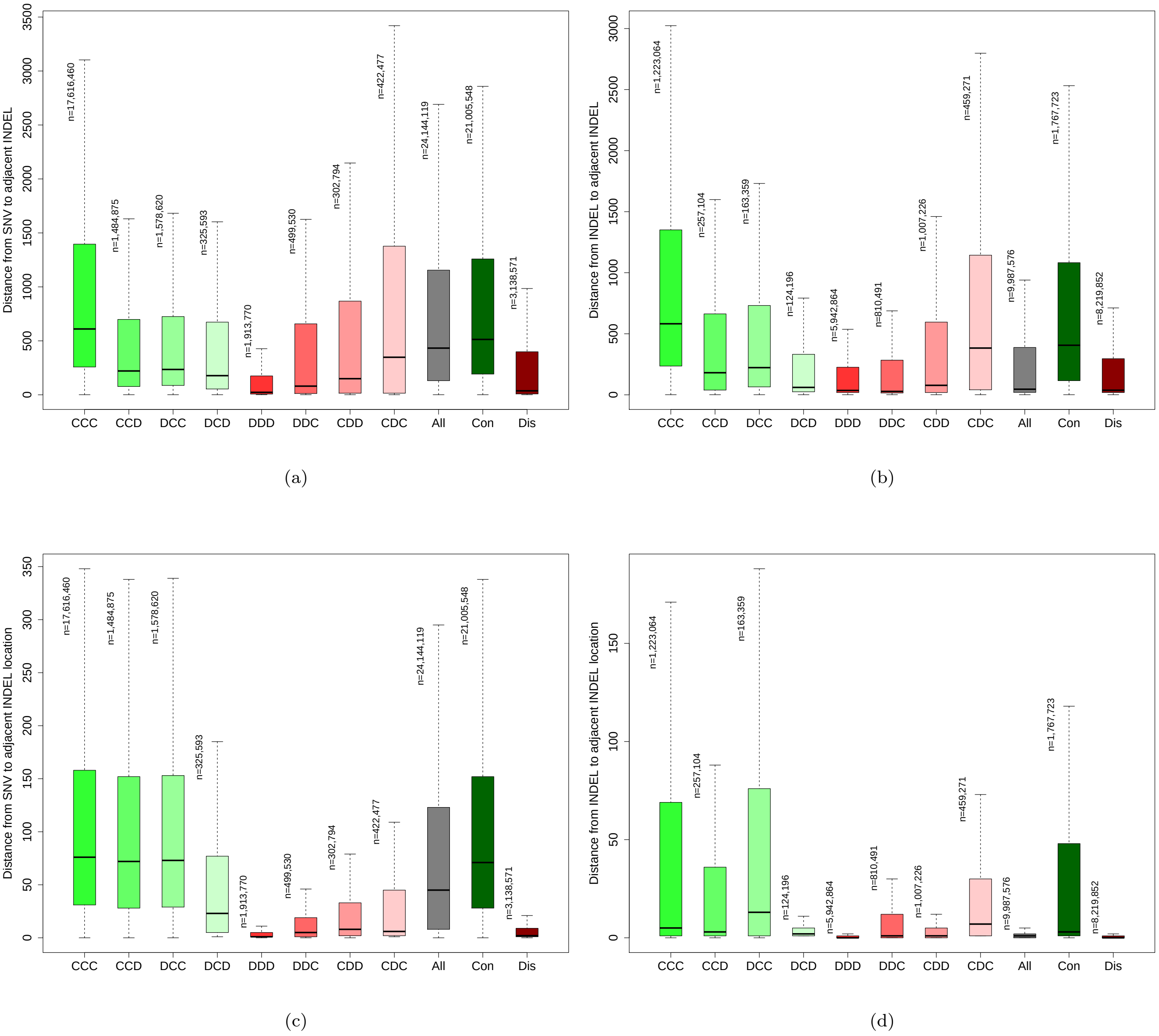

Figure S16: Distance to adjacent INDELs for SNV and INDEL calls. The distance was calculated by taking adjacent INDEL calls within a call set into account (a)+(b) or by taking adjacent INDEL locations in the whole cohort into account (c)+(d). Discordant calls are consistently closer to INDELs which highlights the benefit of realigning/assembling reads around INDELs to reduce false-positive SNV calls (as done by GATK HC and platypus). Note the different scales on the y-axes.

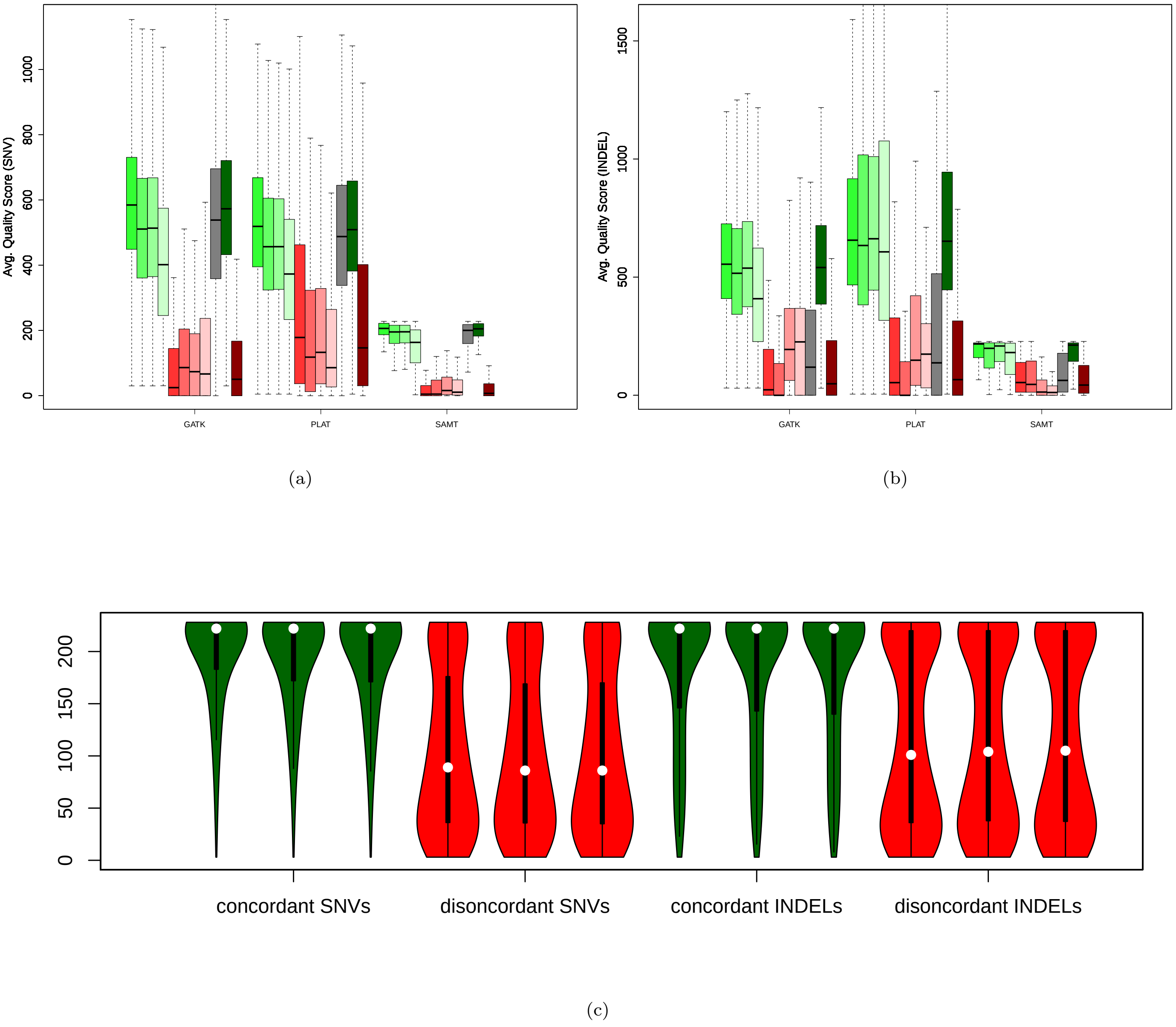

Figure S17: Average quality per variant calling pipeline for SNVs (a) and INDELs (b) and combined violin plots for samtools SNV and INDEL calls of three individual datasets (c). Boxplot colours as in Fig S16. Average variant quality is considerably lower for discordant calls and provides a good though not perfect differentiating factor between concordant and discordant sites. We observed less overlap of the quality value distributions for SNVs; here samtools quality values worked best for our data. For INDELs, averaged quality values still work well as discriminating factors but show more overlap which explains why pure quality-value filtering results in less concordant INDEL calls when compared to SNV calls. Motivated by these results we analysed the possibility to use stringent quality filtering of samtool calls for the prediction of concordance in more detail. However, the violin plots in subfigure (c) that depict the distributions of actual variant quality values of three datasets reveal that there is still considerable overlap between the distributions for concordant and discordant sites, possibly because variant quality is a data-dependent feature that depends largely on mapping and per-base quality values. For this reason it is not recommended to simply rely on stringent quality filtering for concordance prediction. The violin plots were created using the R package *vioplot* by Daniel Adler.

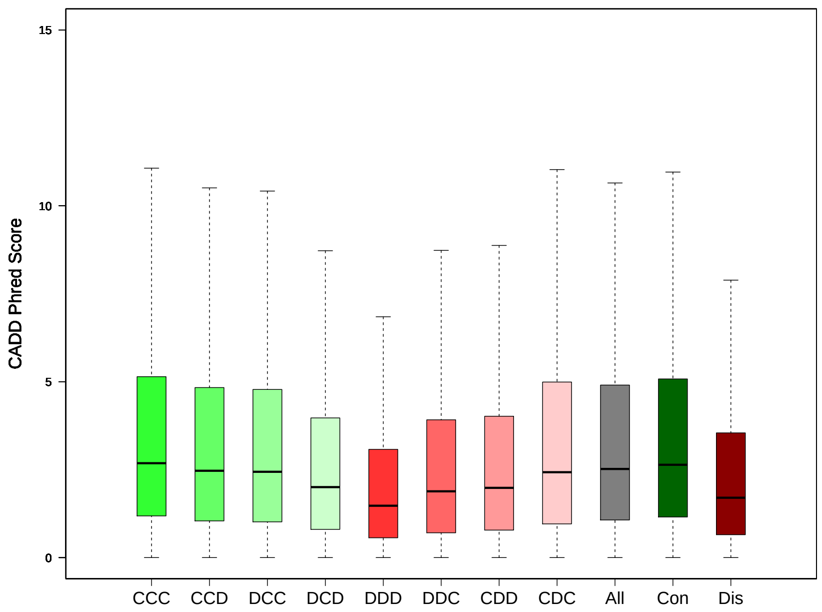

Figure S18: Combined Annotation Dependent Depletion (CADD) [5] phred scores for concordant and discordant SNVs. CADD scores the deleteriousness of variants and our discordant positions show reduced CADD scores when compared to concordant positions.

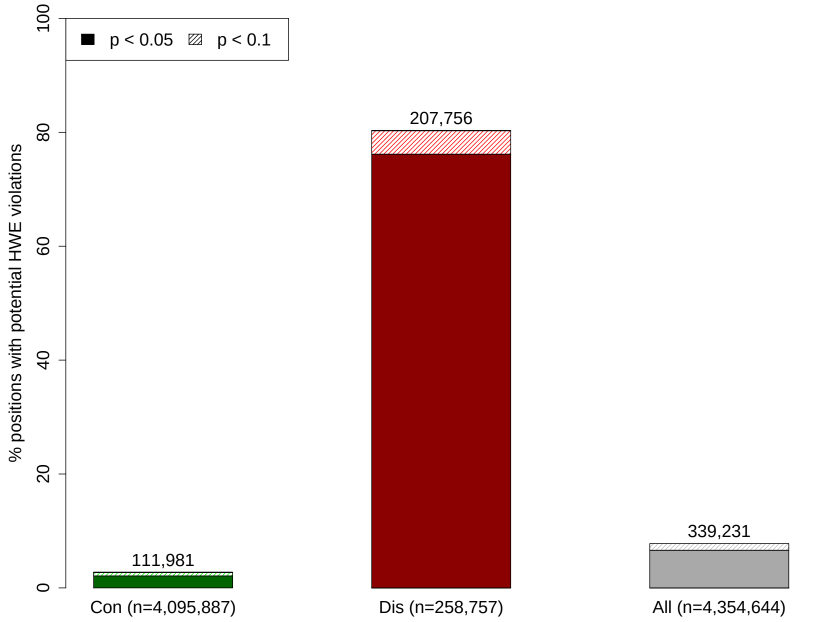

Figure S19: Percentage of concordant/discordant/all biallelic SNVs that potentially violate Hardy-Weinberg Equilibrium (HWE) given the observed genotype calls at two different p-value cutoffs (filled bars: $p_{adj} < 0.05$, dashed bars: $p_{adj} < 0.1$). Only autosomal variants with at least 5 counts in each genotype category were included in this analysis. Genotype counts were averaged over the three variant callsets. P-values were calculated using a χ^2 test (df=1) and adjusted using Benjamini & Hochberg's method. The numbers below the bars show the absolute counts of analysed positions, the numbers above the bars show the absolute numbers of variants with $p_{adj} < 0.1$. Note that a major limitation of the present HWE analysis is that not all of the analysed samples are independent/unrelated and we did not correct for linkage disequilibrium. For this reason, this analysis only has an indicative character and requires further investigation.

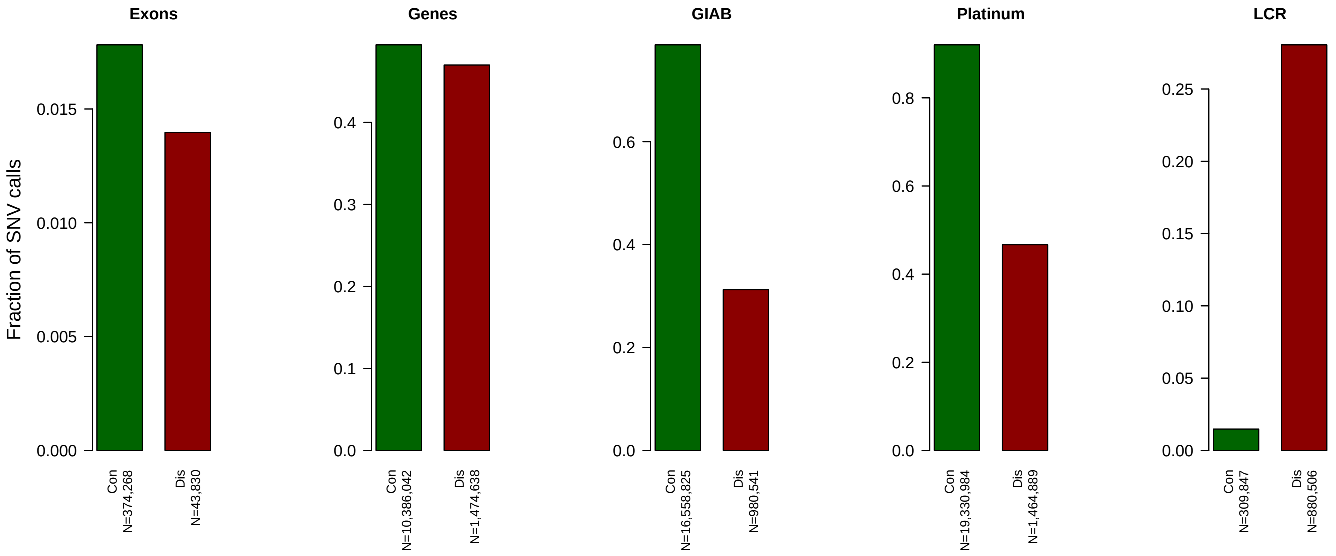

(a)

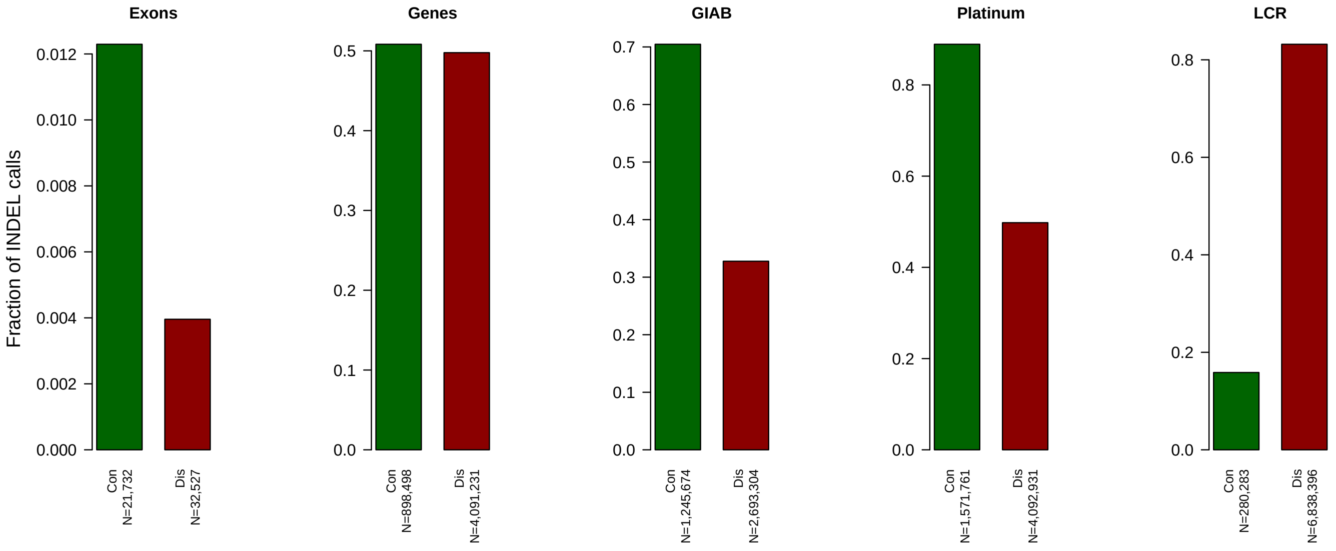

(b)

Figure S20: Distribution of concordant and discordant SNV (a) and INDEL (b) positions between different genomic subregions. Discordant positions are enriched in low-complexity regions and depleted in exonic regions, genes, GIAB concordant regions and Illumina Platinum regions.

6.1 Sequence Context Analysis

Finally, we provide a comprehensive analysis of the reference sequence context around concordant and discordant variant positions. For this analysis, we extracted 21bp windows centred on the called variants from the reference sequence and analysed the sequences with R and FastQC v0.11.2[2]. The following plots are presented in this section:

- Fractions of reference and alternate alleles for SNV calls, Figure S21
- Multi-allelic calls and transition/transversion ratios, Figure S22
- Summary statistics of nucleotide fractions in the analysed sequence contexts, Figure S23
- Sequence context analysis for SNV calls, Figure S24
- Sequence context analysis for INDEL calls, Figure S25
- Sequence context analysis for SNV calls with fixed reference allele, Figure S26
- Sequence context analysis for SNV calls with fixed alternate allele, Figure S27
- Sequence context analysis for $A > N$ SNV calls, Figure S28
- Sequence context analysis for $T > N$ SNV calls, Figure S29
- Sequence context analysis for $C > N$ SNV calls, Figure S30
- Sequence context analysis for $G > N$ SNV calls, Figure S31
- Sequence context analysis for insertions with fixed reference allele, Figure S32

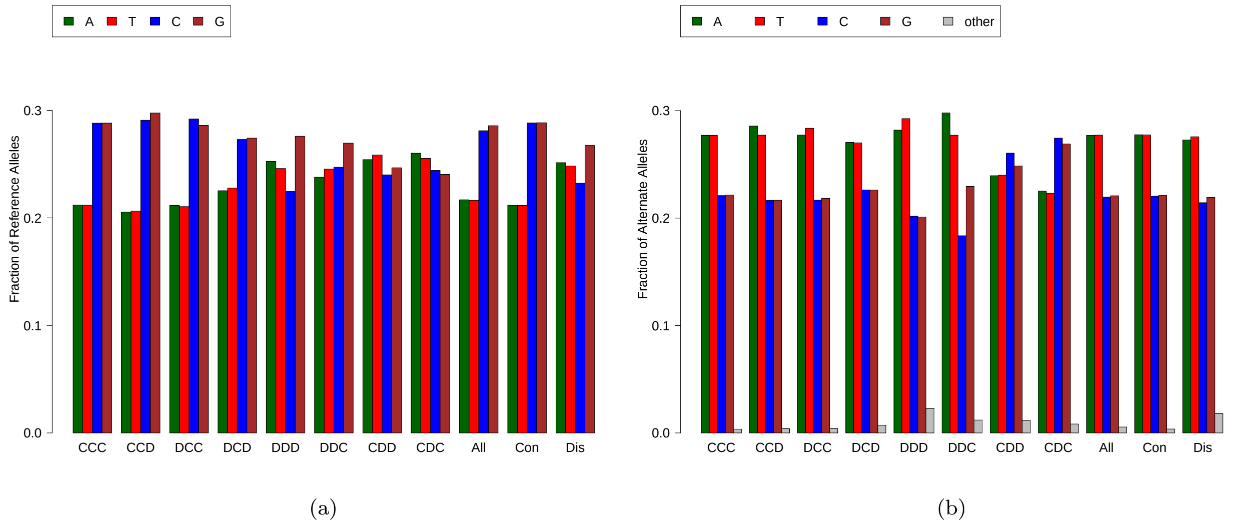

Figure S21: Reference allele percentages (a) and alternate allele percentages (b) for all SNV calls. Multi-allelic calls are plotted in the grey category. The plots show that discordant calls are more likely to have an A or T as reference allele while the alternate allele seems equally distributed as for concordant calls.

[2]http://www.bioinformatics.bbsrc.ac.uk/projects/fastqc/

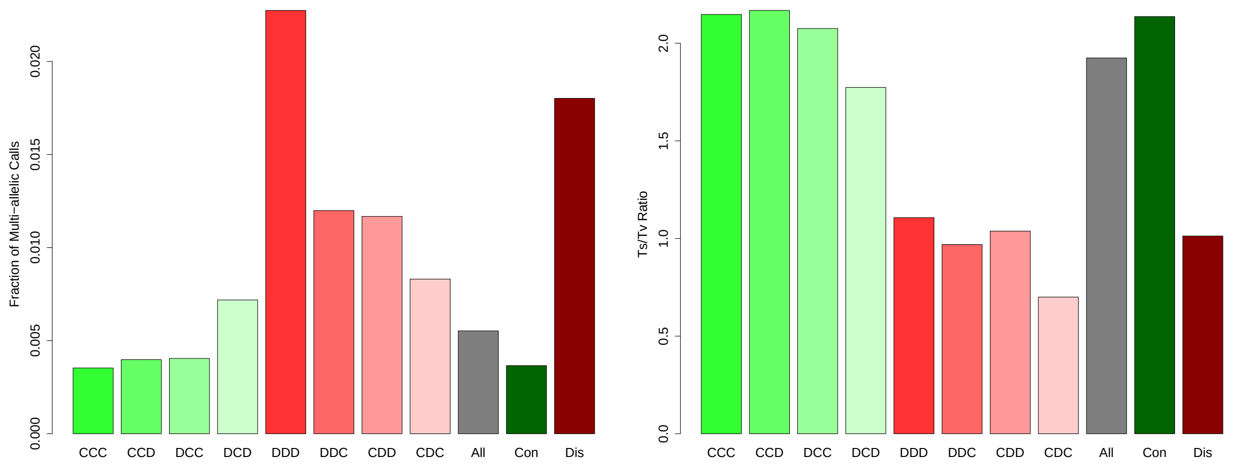

(a) (b)

Figure S22: Percentage of multi-allelic calls (a) and transition/transversion (Ts/Tv) ratios (b) for all SNV calls. Discordant calls are more often multi-allelic and show a decreased Ts/Tv ratio (the expected Ts/Tv for human data is around 2.0-2.1, lower values are often indicators for sequencing artefacts, cf. [10]).

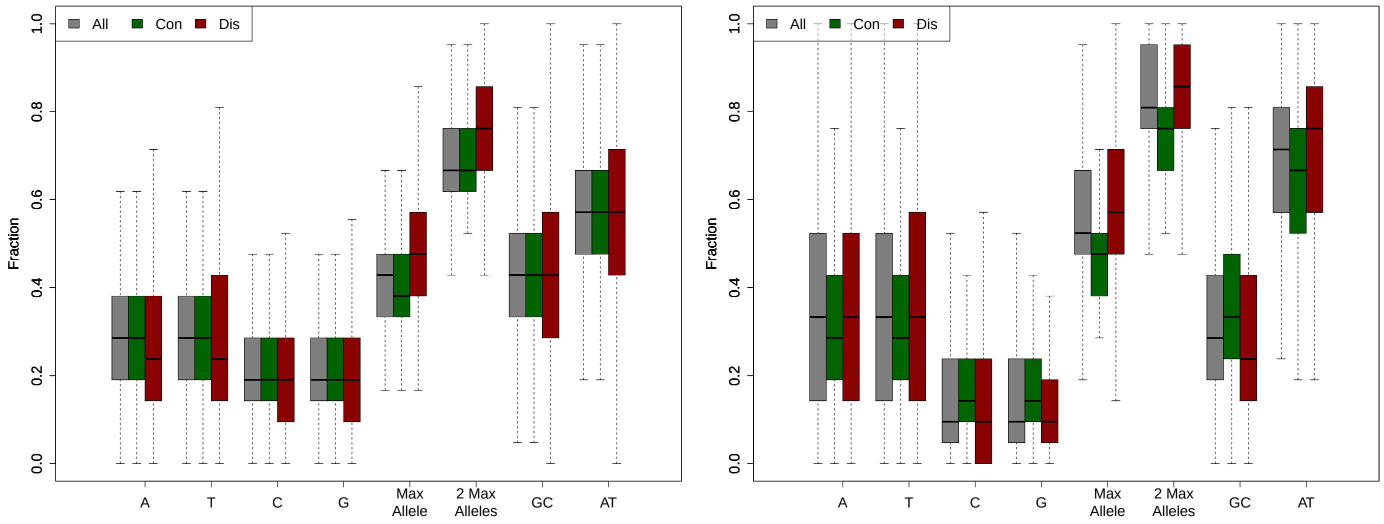

(a) (b)

Figure S23: Fractions of nucleotides in a 21 bp window centered on the called positions for (a) SNVs and (b) INDELs. Max allele: fraction of most frequent nucleotide in context; 2 Max Alleles: sum of fractions of the two most frequent nucleotides; GC/AT: sums of fractions of the respective nucleotides (please note that this fraction can be below 0.5 for multi-allelic calls). The data reveals decreased sequence complexity in the context of discordant positions (see the “Max Allele(s)” categories) which is even more pronounced for INDELs and an AT-enrichment in the sequence context for discordant INDELs but not for SNVs. Note that it was previously reported that Illumina data contains systematic errors around poly-A regions [7] and that such regions would be strongly enriched in LCR regions.

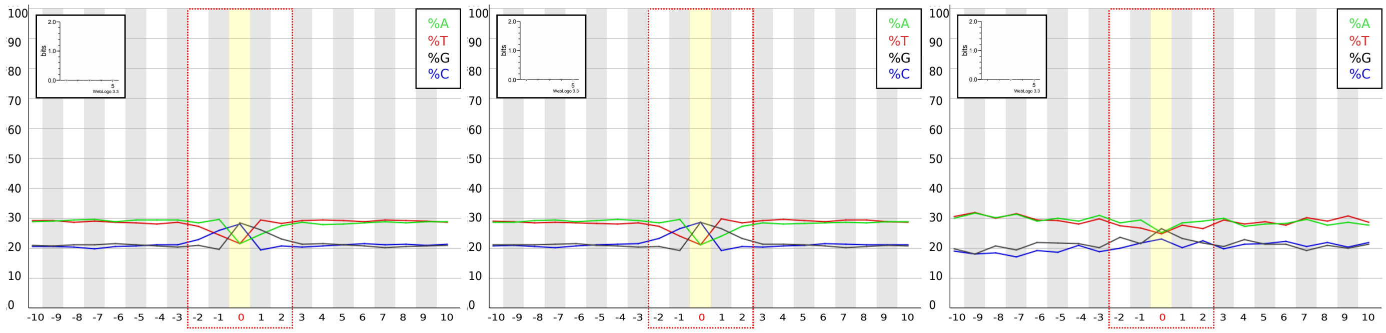

(a) All SNVs, n=24,007,773 (b) Con SNVs, n=20,928,670 (c) Dis SNVs, n=3,079,103

Figure S24: Sequence context around (a) all, (b) concordant, and (c) discordant SNV calls. The y-axes show the percentages of the respective bases (A: green, T: red, G: black, C:blue) in the 21bp sequence context around the variant (at position 0, highlighted in yellow). The 5bp nucleotide context [-2, +2] is highlighted by a dashed red border and a sequence logo[2] of this context is provided in the upper left corner of the plot. The numbers of considered sequences are printed in the subfigure captions, incomplete sequence context (containing N characters) and sequences from multi-allelic calls were not considered in this analysis. The percentage curves in (a) and (b) show a clear bias inside a context of 5-7 bases around the variant which is consistent with the findings in [1, 16]. The signal outside of this context is consistent with the expected GC percentage in the human genome (%GC∼40.45, [1]). The signal for discordant positions in (c) is clearly distorted and the observable zigzag pattern indicates an enrichment in low-complexity regions/dinucleotide microsatellite regions around discordant positions.

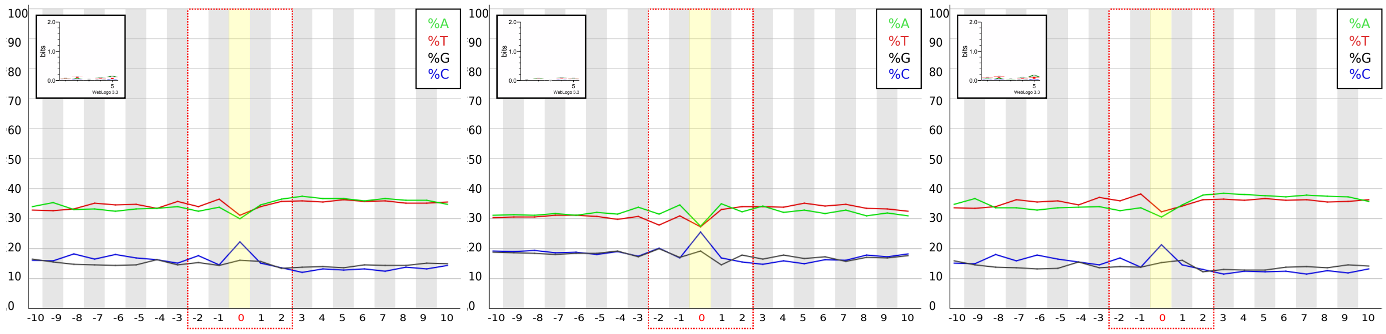

(a) All INDELs, n=7,670,650 (b) Con INDELs, n=1,713,958 (c) Dis INDELs, n=5,956,692

Figure S25: Sequence context around (a) all, (b) concordant, and (c) discordant INDEL calls. See Figure S24 for a general description of the plots. The base at position 0 corresponds to the leftmost reference position of the INDEL as provided in the VCF files. INDELs are generally called in more AT-rich regions which is pronounced in discordant positions. A zigzag pattern indicating low-complexity sequences can be observed in both, concordant and discordant positions. While concordant variants show a slight enrichment in A's left of the variants, discordant variants seem to be enriched in T's at the same relative position.

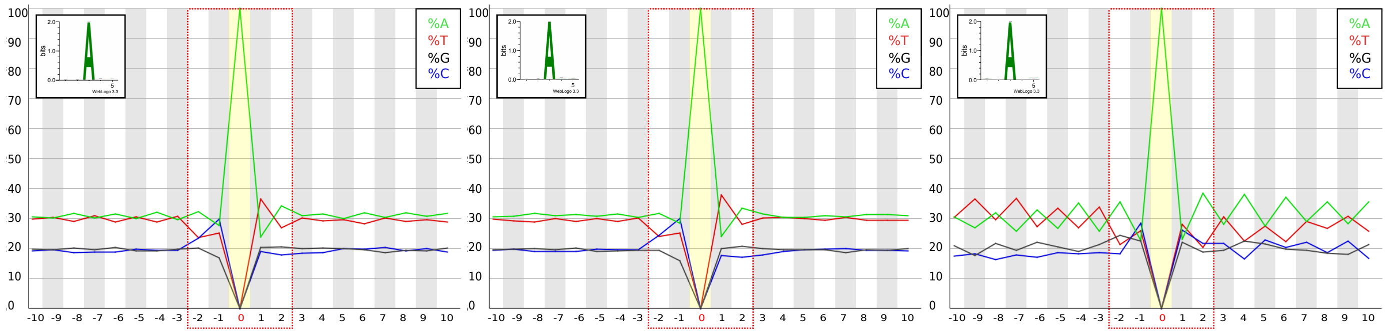

(a) All $A > N$ SNVs, n=5,210,465 (b) Con $A > N$ SNVs, n=4,433,897 (c) Dis $A > N$ SNVs, n=776,568

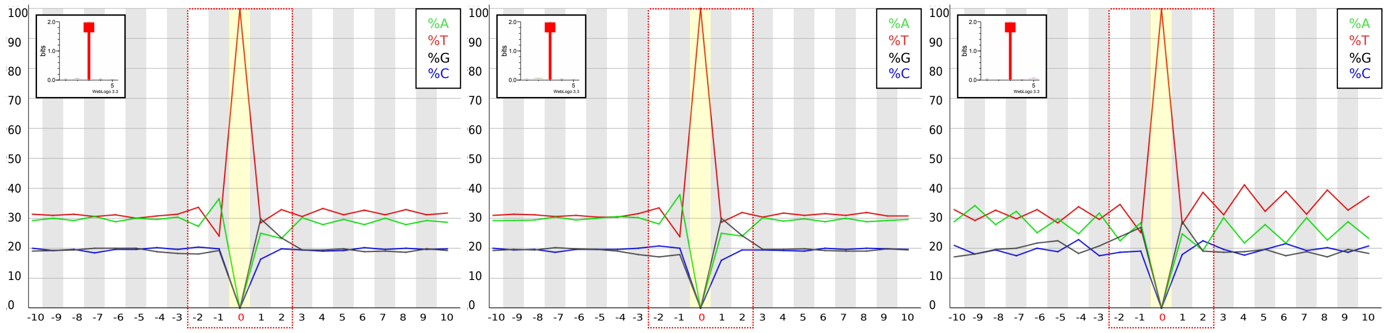

(d) All $T > N$ SNVs, n=5,199,203 (e) Con $T > N$ SNVs, n=4,432,253 (f) Dis $T > N$ SNVs, ,n=766950

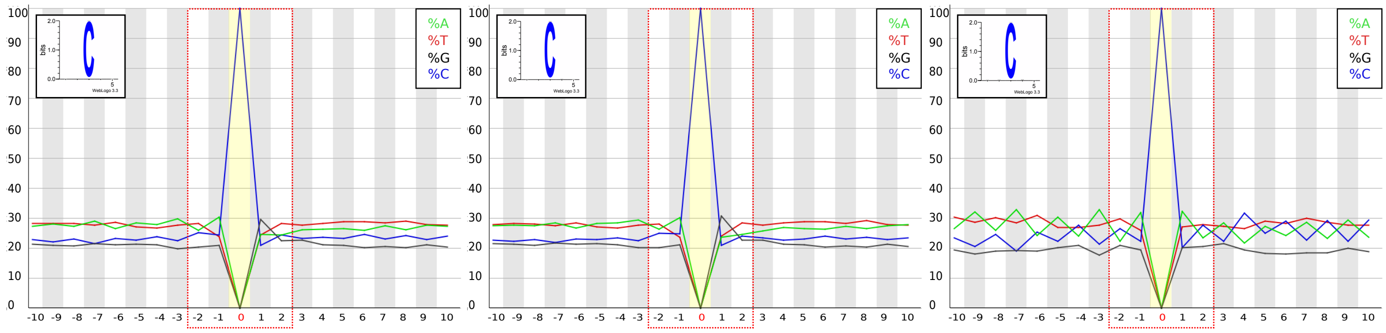

(g) All $C > N$ SNVs, n=6,743,246 (h) Con $C > N$ SNVs, n=6,030,112 (i) Dis $C > N$ SNVs, n=713,134

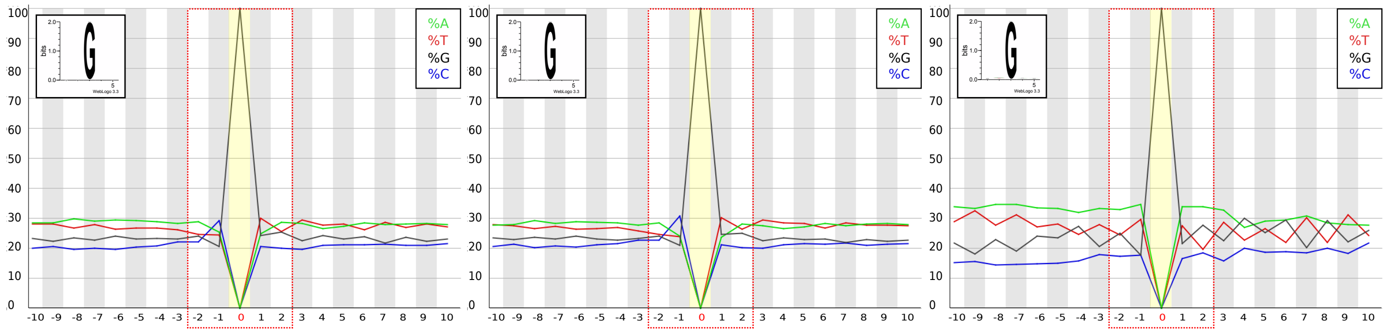

(j) All $G > N$ SNVs, n=6,854,859 (k) Con $G > N$ SNVs, n=6,032,408 (l) Dis $G > N$ SNVs, n=822,451

Figure S26: Sequence context around SNV calls with fixed *reference* allele: A (plots a-c), T (plots d-f), C (plots g-i), G (plots j-l). The subplots show the signals for all (1st column), concordant (2nd column) and discordant (3rd column) calls. See Figure S24 for a general description of the plots. Note the clear symmetry for the concordant A/T and G/C plots respectively which is much less pronounced for the discordant calls. Again, the plots for the discordant variants (3rd column) clearly show the previously discussed zigzag pattern and seem significantly distorted when compared to the concordant ones.

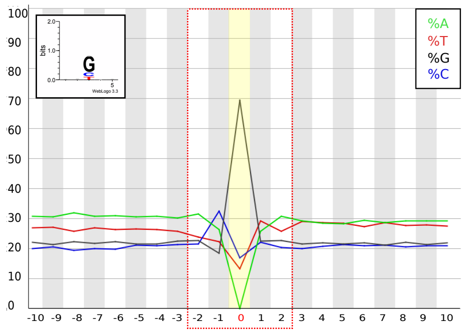

(a) All $N > A$ SNVs, n=6,685,039

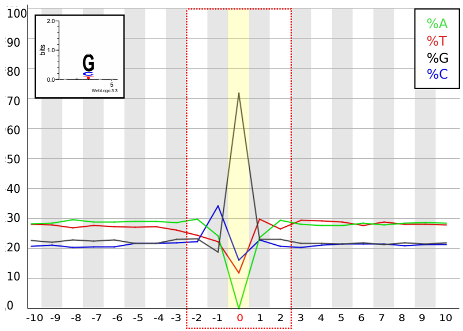

(b) Con $N > A$ SNVs, n=5,829,952

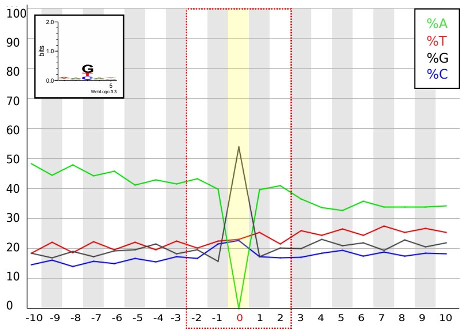

(c) Dis $N > A$ SNVs, n=855,087

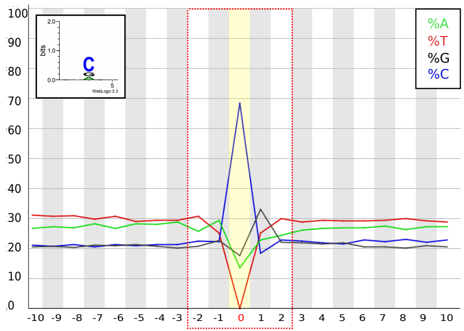

(d) All $N > T$ SNVs, n=6,692,334

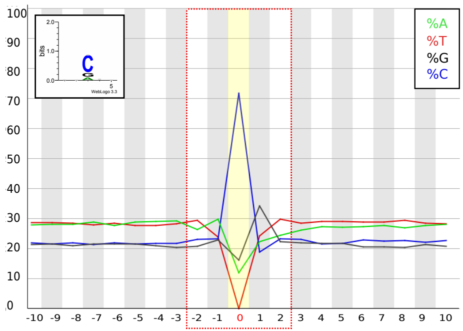

(e) Con $N > T$ SNVs, n=5,827,901

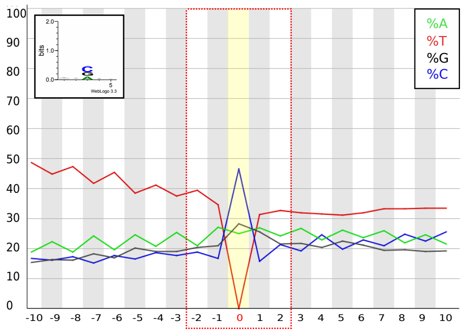

(f) Dis $N > T$ SNVs, n=864,433

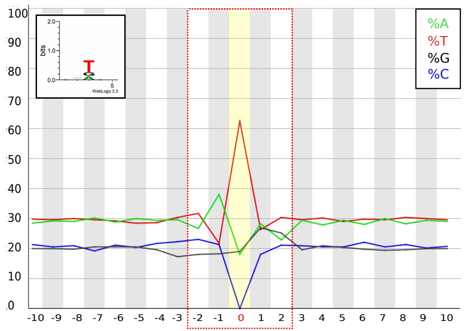

(g) All $N > C$ SNVs, n=5,301,151

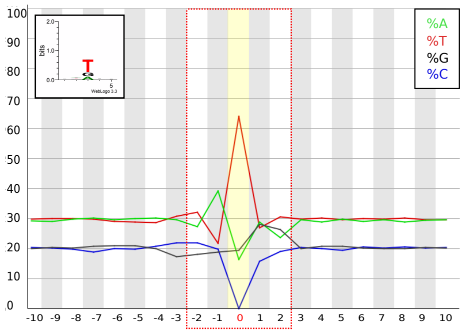

(h) Con $N > C$ SNVs, ,n=4,629,010

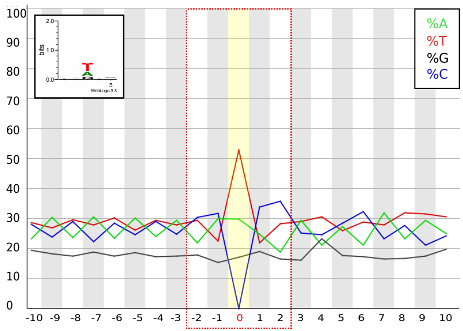

(i) Dis $N > C$ SNVs, n=672,141

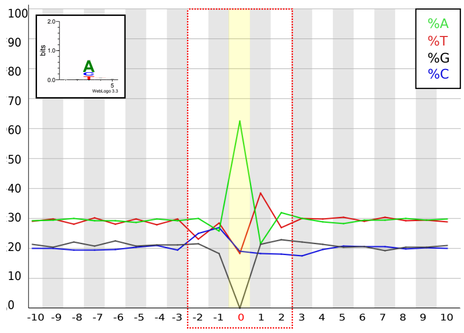

(j) All $N > G$ SNVs, n=5,329,249

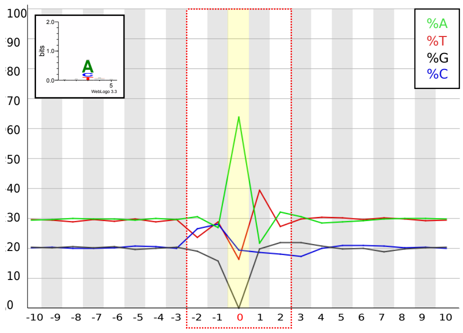

(k) Con $N > G$ SNVs, n=4,641,807

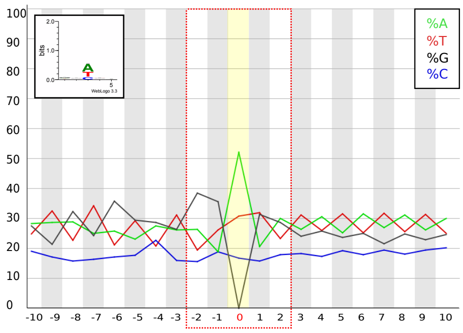

(l) Dis $N > G$ SNVs, n=687,442

Figure S27: Sequence context analysis of SNV calls with fixed *alternate* allele: A (plots a-c), T (plots d-f), C (plots g-i), G (plots j-l). See Figure S24 for a general description of the plots. The sequence contexts for discordant $N > A$ and $N > T$ variants are highly enriched for (poly-)A/T sequences. Note that for all discordant sequences, the found percentages for the alternate alleles seem well below the expected Ts/Tv ratio of around 2.1 (alternate alleles resulting from a transition would be expected to contribute about 70%).

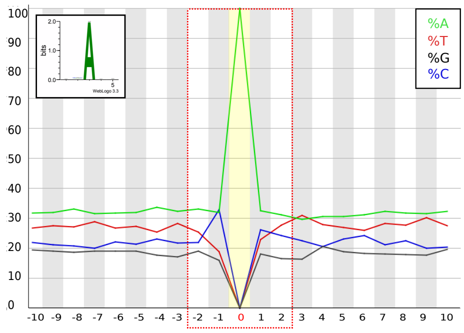

(a) All $A > C$ SNVs, n=963,081

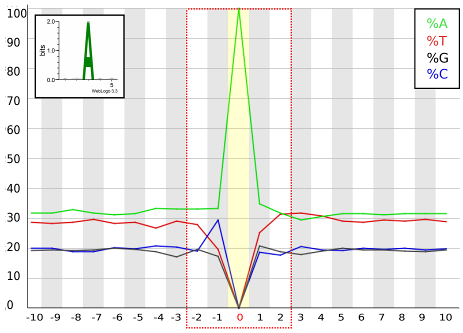

(b) Con $A > C$ SNVs, n=762,081

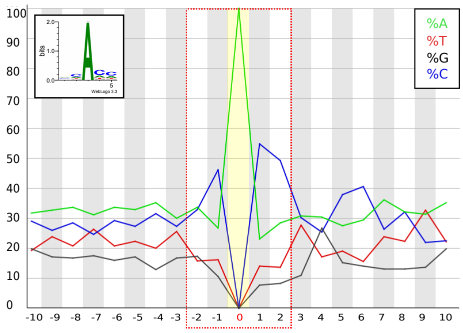

(c) Dis $A > C$ SNVs, n=201,000

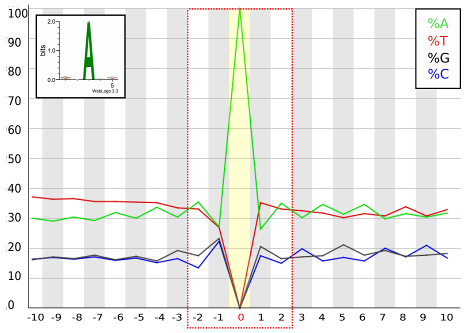

(d) All $A > T$ SNVs, n=916,224

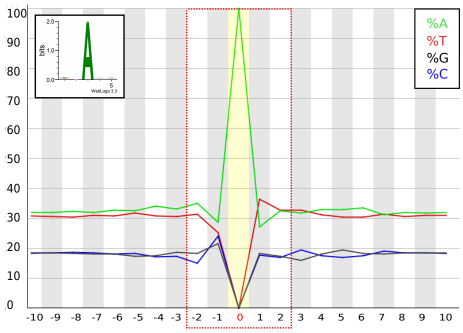

(e) Con $A > T$ SNVs, n=699,913

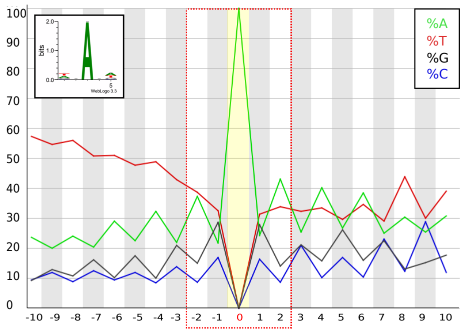

(f) Dis $A > T$ SNVs, n=216,311

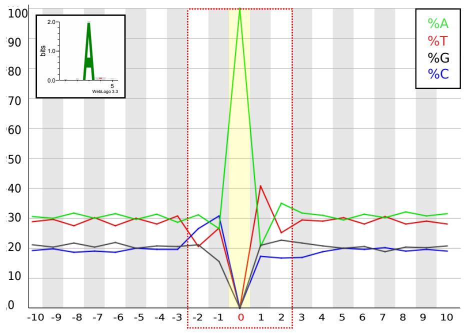

(g) All $A > G$ SNVs, n=3,331,160

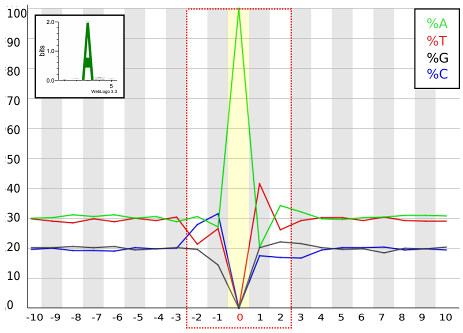

(h) Con $A > G$ SNVs, n=2,971,903

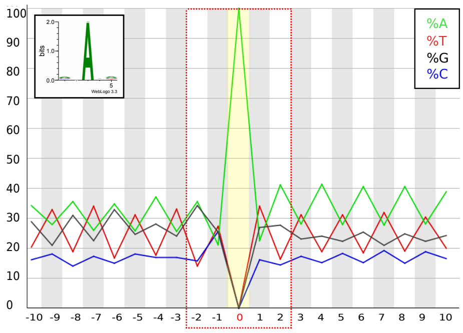

(i) Dis $A > G$ SNVs, n=359,257

Figure S28: Sequence context analysis of $A > N$ SNVs. See Figure S24 for a general description of the plots. Note that our results for concordant variants are consistent with the increased substitution probabilities of ApT dinucleotides recently reported in [1]. There, the authors report increased $ApT > GpT$ and $ApT > TpT$ substitution rates which correspond to the T-peaks next to the $A > G$ and $A > T$ variants in the plots above. Plot (f) shows that discordant $A > T$ variants are called often at the right end of (poly-)T enriched regions.

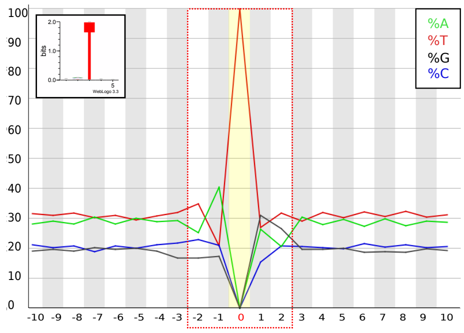

(a) All $T > C$ SNVs, n=3,322,578

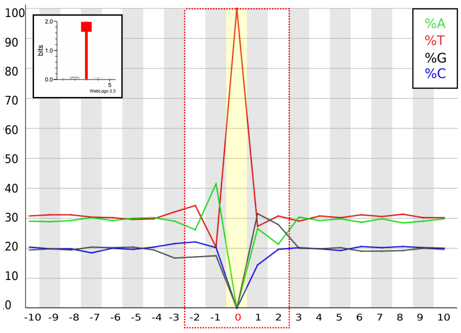

(b) Con $T > C$ SNVs, n=2,966,901

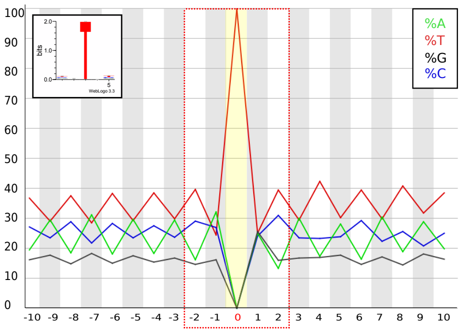

(c) Dis $T > C$ SNVs, n=355,677

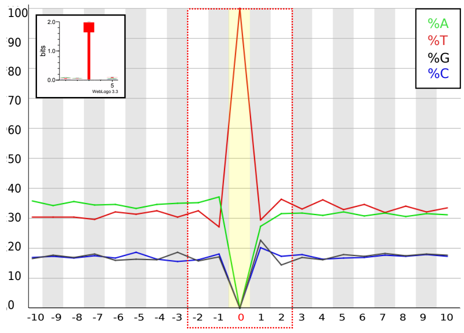

(d) All $T > A$ SNVs, n=897,469

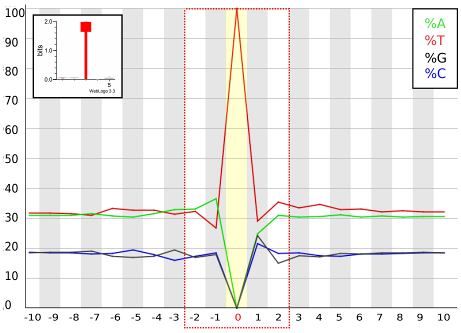

(e) Con $T > A$ SNVs, n=698,840

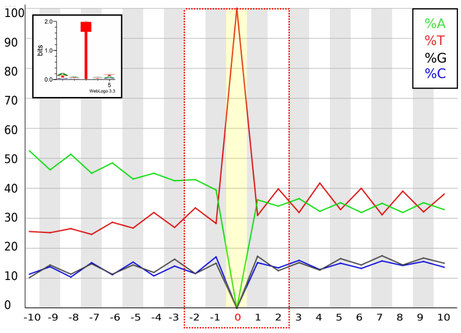

(f) Dis $T > A$ SNVs, n=198,629

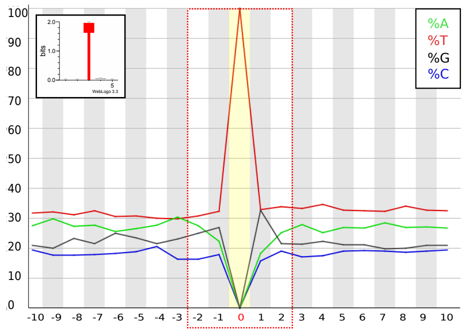

(g) All $T > G$ SNVs, n=979,156

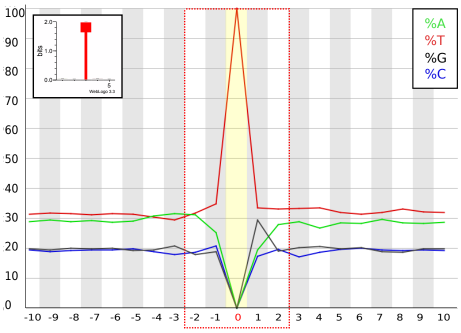

(h) Con $T > G$ SNVs, n=766,512

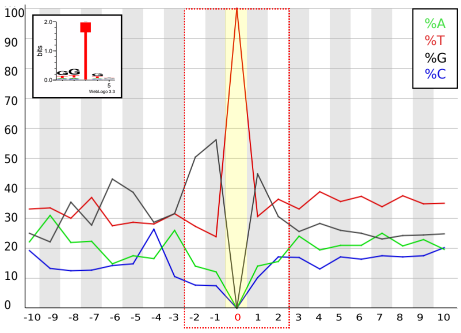

(i) Dis $T > G$ SNVs, n=212,644

Figure S29: Sequence context analysis of $T > N$ SNVs. See Figure S24 for a general description of the plots. The plots are largely symmetric to the $A > N$ plots discussed above. A notable exception can be seen in plot (f) that shows that discordant $T > A$ are called at the right (not left) end of (poly-)A enriched regions (compare with plot (f) in FigureS28).

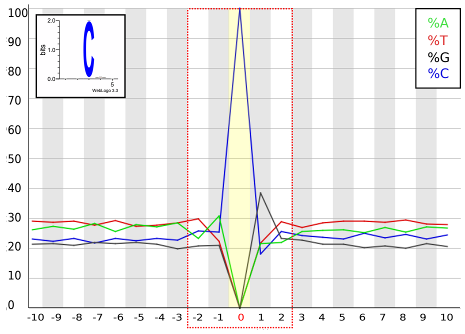

(a) All $C > T$ SNVs, n=4,585,583

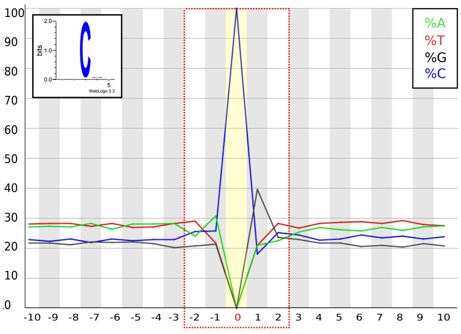

(b) Con $C > T$ SNVs, n=4,182,837

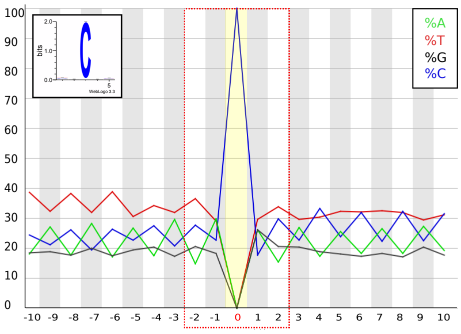

(c) Dis $C > T$ SNVs, n=402,746

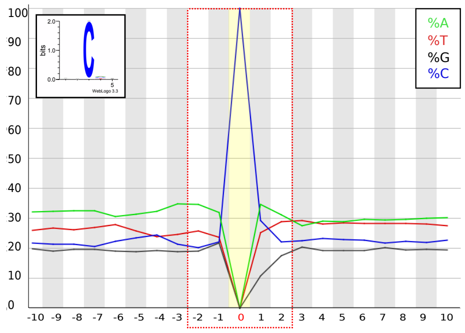

(d) All $C > A$ SNVs, n=1,138,730

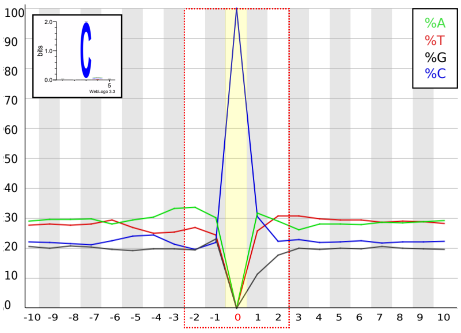

(e) Con $C > A$ SNVs, n=943,883

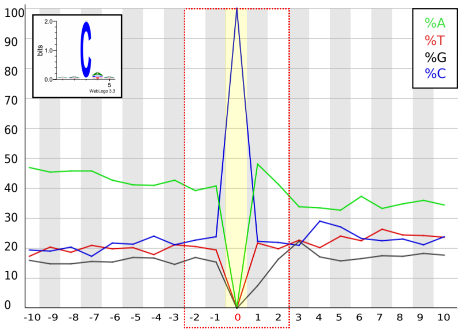

(f) Dis $C > A$ SNVs, n=194,847

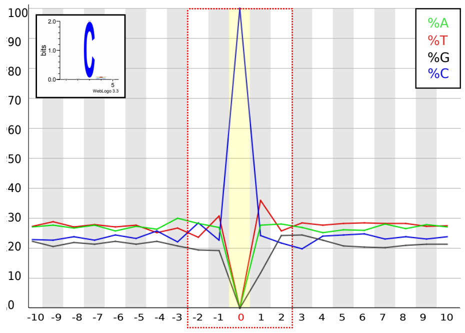

(g) All $C > G$ SNVs, n=1,018,933

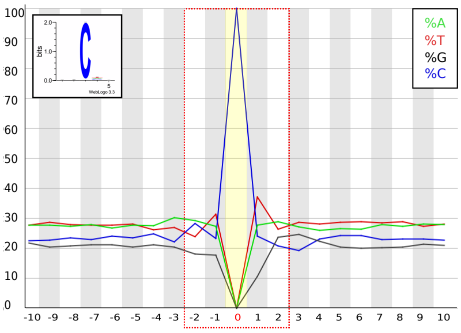

(h) Con $C > G$ SNVs, n=903,392

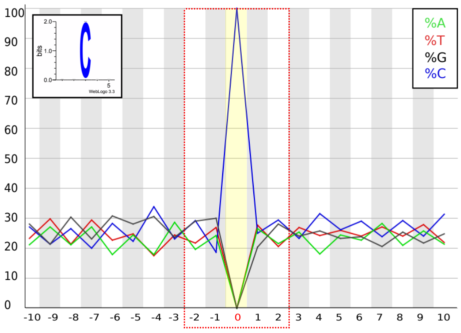

(i) Dis $C > G$ SNVs, n=115,541

Figure S30: Sequence context analysis of $C > N$ SNVs. See Figure S24 for a general description of the plots. Note that the observed pattern is consistent with the known high mutability of methylated CpG's ($C^m pG \rightarrow TG$), see G-peak next to the $C > T$ variants. In humans, about 80% of the CpG's are methylated, [18]. Notably, discordant $C > A$ are called in regions that are highly enriched in A's. Generally, discordant $N > A$ and $N > T$ calls are found in enriched A/T regions.

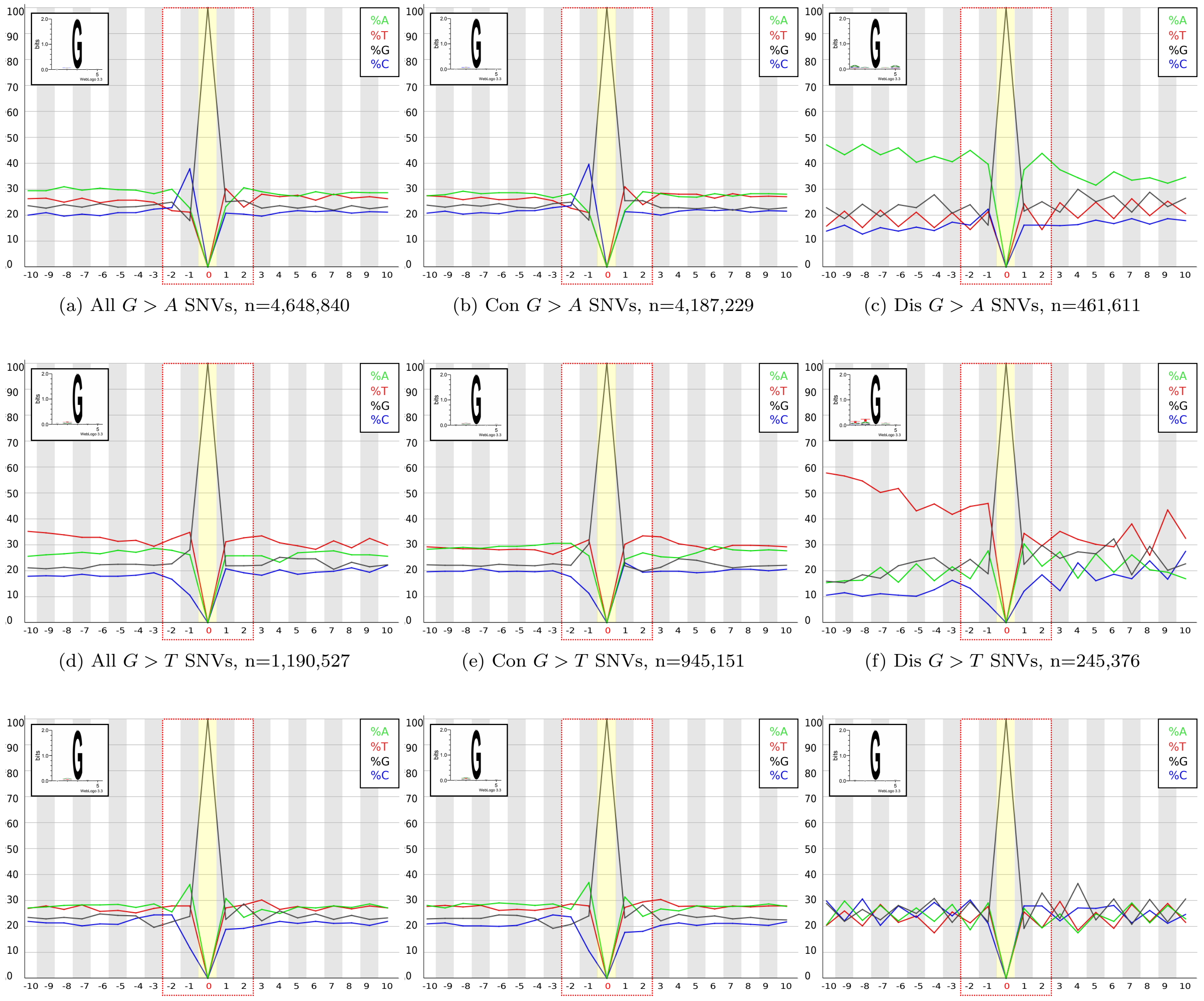

(a) All $G > A$ SNVs, n=4,648,840 (b) Con $G > A$ SNVs, n=4,187,229 (c) Dis $G > A$ SNVs, n=461,611

(d) All $G > T$ SNVs, n=1,190,527 (e) Con $G > T$ SNVs, n=945,151 (f) Dis $G > T$ SNVs, n=245,376

(g) All $G > C$ SNVs, n=1,015,492 (h) Con $G > C$ SNVs, n=900,028 (i) Dis $G > C$ SNVs, n=115,464

Figure S31: Sequence context analysis of $G > N$ SNVs. See Figure S24 for a general description of the plots. Note the symmetry with the $C > N$ plots in Figure S30.

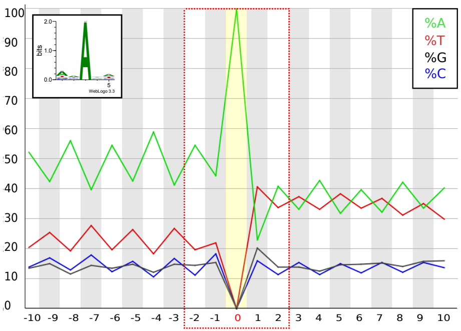

(a) All $A > N+$ insertions, n=641,024

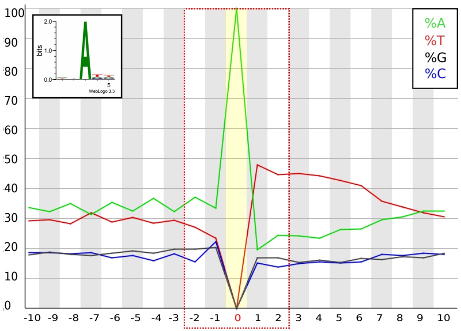

(b) Con $A > N+$ insertions, n=143,358

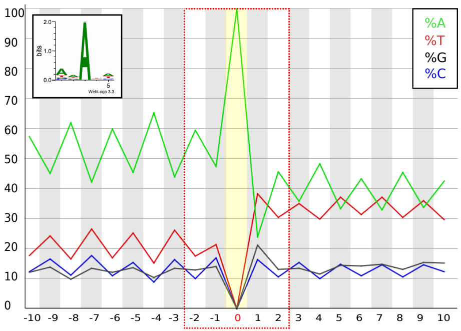

(c) Dis $A > N+$ insertions, n=497,666

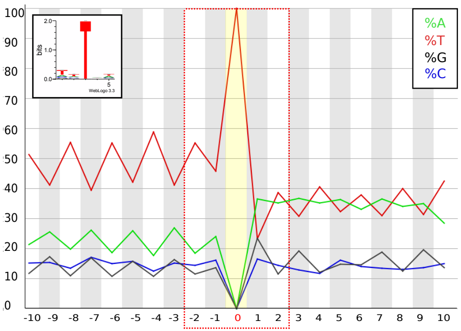

(d) All $T > N+$ insertions, n=640,716

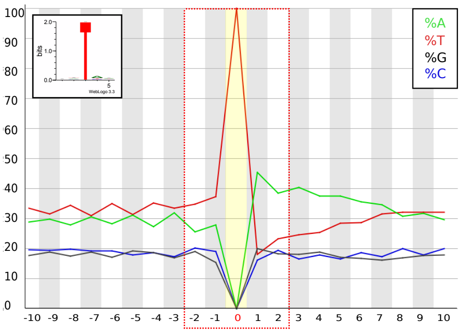

(e) Con $T > N+$ insertions, n=143,631

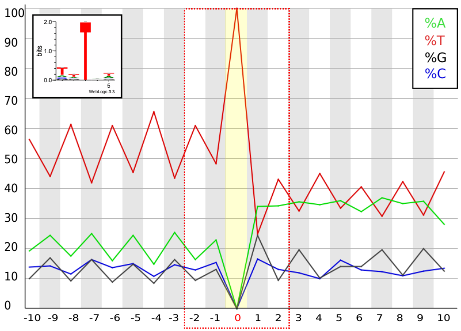

(f) Dis $T > N+$ insertions, n=497,085

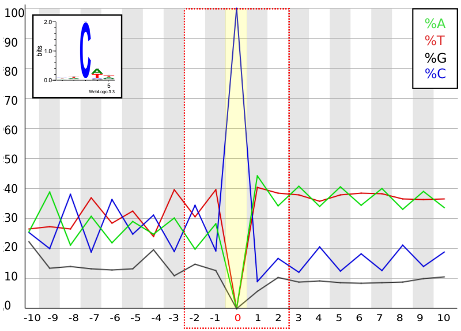

(g) All $C > N+$ insertions, n=523,496

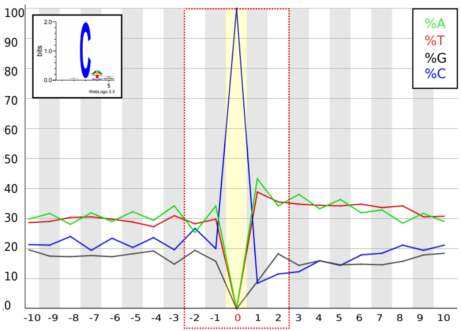

(h) Con $C > N+$ insertions, n=135,171

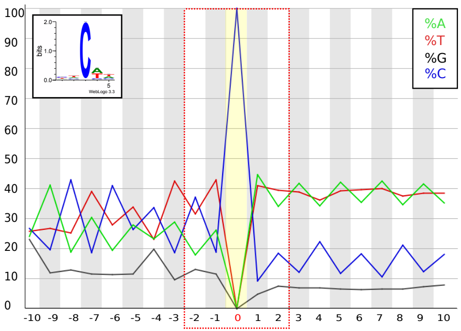

(i) Dis $C > N+$ insertions, n=388,325

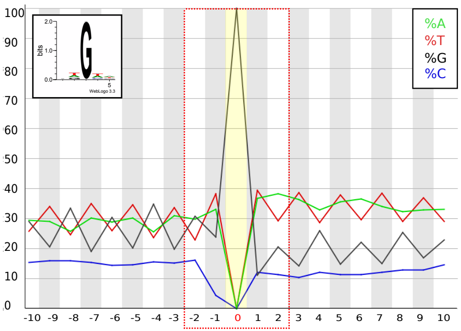

(j) All $G > N+$ insertions, n=420,045

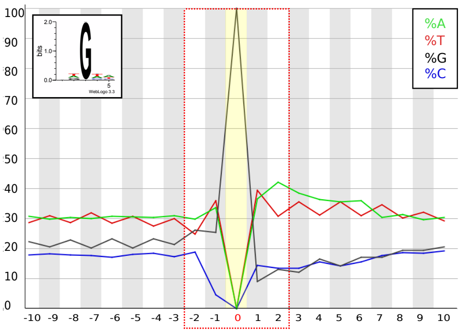

(k) Con $G > N+$ insertions, n=121,122

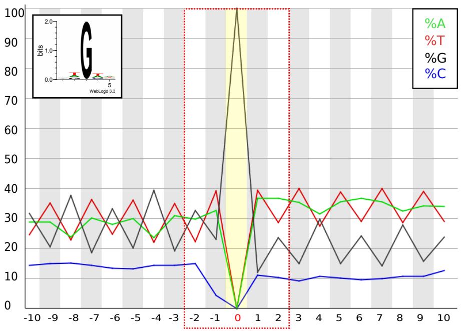

(l) Dis $G > N+$ insertions, n=298,923

Figure S32: Sequence context analysis for insertions with fixed reference allele. See Figure S24 for a general description of the plots. As in the plots above, the pronounced zigzag pattern around discordant calls indicates low sequence-complexity.

7 Authors' contributions

NP designed and conducted the experiments, wrote the code, analysed the data and wrote the paper. AS and JT helped develop the concept of RG, identified its use case and potential applications and edited the paper. JT directed the research. The WGS500 Consortium provided the raw data for the main experiments. A list of WGS500 Consortium members and affiliations is provided below. All authors approved the final version of the manuscript.

7.1 WGS500 Consortium

Steering Committee. Peter Donnelly[1] (Chair), John Bell[2], David Bentley[3], Gil McVean[1], Peter Ratcliffe[1], Jenny Taylor[1,4], Andrew Wilkie[4,5]

Operations Committee. Peter Donnelly[1] (Chair), John Broxholme[1], David Buck[1], Jean-Baptiste Cazier[1], Richard Cornall[1], Lorna Gregory[1], Julian Knight[1], Gerton Lunter[1], Gilean McVean[1], Jenny Taylor[1,4], Ian Tomlinson[1,4], Andrew Wilkie[4,5]

Sequencing & Experimental Follow Up. David Buck[1] (Lead), Christopher Allan[1], Moustafa Attar[1], Angie Green[1], Lorna Gregory[1], Sean Humphray[3], Zoya Kingsbury[3], Sarah Lamble[1], Lorne Lonie[1], Alistair Pagnamenta[1,4], Paolo Piazza[1], Guadelupe Polanco[1], Amy Trebes[1]

Data Analysis. Gil McVean[1] (Lead), Peter Donnelly[1], Jean-Baptiste Cazier[1], John Broxholme[1], Richard Copley[1], Simon Fiddy[1], Russell Grocock[3], Edouard Hatton[1], Chris Holmes[1], Linda Hughes[1], Peter Humburg[1], Alexander Kanapin[1], Stefano Lise[1], Gerton Lunter[1], Hilary Martin[1], Davis McCarthy[1], Lisa Murray[3], Andy Rimmer[1], Natasha Sahgal[1], Ben Wright[1], Chris Yau[6]

[1] *The Wellcome Trust Centre for Human Genetics, Roosevelt Drive, Oxford, OX3 7BN, UK.*
[2] *Office of the Regius Professor of Medicine, Richard Doll Building, Roosevelt Drive, Oxford, OX3 7LF, UK.*
[3] *Illumina Cambridge Ltd., Chesterford Research Park, Little Chesterford, Essex, CB10 1XL, UK.*
[4] *NIHR Oxford Biomedical Research Centre, Oxford, UK.*
[5] *Weatherall Inst of Molecular Medicine, University of Oxford; John Radcliffe Hospital Headington, Oxford OX3 9DS, UK.*
[6] *Imperial College London, South Kensington Campus, London, SW7 2AZ, UK.*

8 Supplemental References

References

[1] V. Aggarwala and B. F. Voight. An expanded sequence context model broadly explains variability in polymorphism levels across the human genome. *Nat Genet*, 48(4):349–355, Apr. 2016.

[2] G. E. Crooks, G. Hon, J.-M. Chandonia, and S. E. Brenner. Weblogo: a sequence logo generator. *Genome Res*, 14(6):1188–1190, Jun 2004.

[3] M. A. DePristo, E. Banks, R. Poplin, K. V. Garimella, J. R. Maguire, C. Hartl, A. A. Philippakis, G. del Angel, M. A. Rivas, M. Hanna, A. McKenna, T. J. Fennell, A. M. Kernytsky, A. Y. Sivachenko, K. Cibulskis, S. B. Gabriel, D. Altshuler, and M. J. Daly. A framework for variation discovery and genotyping using next-generation dna sequencing data. *Nat Genet*, 43(5):491–498, May 2011.

[4] E. Garrison. https://github.com/ekg/vcflib. 2015.

[5] M. Kircher, D. M. Witten, P. Jain, B. J. O'Roak, G. M. Cooper, and J. Shendure. A general framework for estimating the relative pathogenicity of human genetic variants. *Nat Genet*, 46(3):310–315, Mar 2014.

[6] H. Li. Toward better understanding of artifacts in variant calling from high-coverage samples. *Bioinformatics*, 30(20):2843–2851, Oct 2014.

[7] H. Li. Fermikit: assembly-based variant calling for illumina resequencing data. *Bioinformatics*, 31(22):3694–3696, Nov 2015.

[8] H. Li and R. Durbin. Fast and accurate short read alignment with burrows-wheeler transform. *Bioinformatics*, 25(14):1754–1760, Jul 2009.

[9] H. Li, B. Handsaker, A. Wysoker, T. Fennell, J. Ruan, N. Homer, G. Marth, G. Abecasis, R. Durbin, and the 1000 Genome Project Data Processing Subgroup. The sequence alignment/map format and samtools. *Bioinformatics*, 25(16):2078–2079, Aug 2009.

[10] Q. Liu, Y. Guo, J. Li, J. Long, B. Zhang, and Y. Shyr. Steps to ensure accuracy in genotype and snp calling from illumina sequencing data. *BMC Genomics*, 13 Suppl 8:S8, 2012.

[11] G. Lunter and M. Goodson. Stampy: a statistical algorithm for sensitive and fast mapping of illumina sequence reads. *Genome Res*, 21(6):936–939, Jun 2011.

[12] N. Popitsch. Codoc: efficient access, analysis and compression of depth of coverage signals. *Bioinformatics*, 30(18):2676–2677, Sep 2014.

[13] N. Popitsch, P. Rescheneder, and M. Gallach. Intragenomic sequence similarity patterns assist in de novo genome annotation. In *Poster at NGS'14*, 2014.

[14] C. Raczy, R. Petrovski, C. T. Saunders, I. Chorny, S. Kruglyak, E. H. Margulies, H.-Y. Chuang, M. Källberg, S. A. Kumar, A. Liao, K. M. Little, M. P. Strömberg, and S. W. Tanner. Isaac: ultra-fast whole-genome secondary analysis on illumina sequencing platforms. *Bioinformatics*, 29(16):2041–2043, Aug 2013.

[15] A. Rimmer, H. Phan, I. Mathieson, Z. Iqbal, S. R. F. Twigg, the WGS500 Consortium, A. O. M. Wilkie, G. McVean, and G. Lunter. Integrating mapping-, assembly- and haplotype-based approaches for calling variants in clinical sequencing applications. *Nat Genet*, 46(8):912–918, Aug 2014.

[16] A. B. Sahakyan and S. Balasubramanian. Core variability in substitution rates and the basal sequence characteristics of the human genome. *bioRxiv*, 2016.

[17] J. C. Taylor, H. C. Martin, S. Lise, J. Broxholme, J.-B. Cazier, A. Rimmer, A. Kanapin, G. Lunter, S. Fiddy, C. Allan, A. R. Aricescu, M. Attar, C. Babbs, J. Becq, D. Beeson, C. Bento, P. Bignell, E. Blair, V. J. Buckle, K. Bull, O. Cais, H. Cario, H. Chapel, R. R. Copley, R. Cornall, J. Craft, K. Dahan, E. E. Davenport, C. Dendrou, O. Devuyst, A. L. Fenwick, J. Flint, L. Fugger, R. D. Gilbert, A. Goriely, A. Green, I. H. Greger, R. Grocock, A. V. Gruszczyk, R. Hastings, E. Hatton, D. Higgs, A. Hill, C. Holmes, M. Howard, L. Hughes, P. Humburg, D. Johnson, F. Karpe, Z. Kingsbury, U. Kini, J. C. Knight, J. Krohn, S. Lamble, C. Langman, L. Lonie, J. Luck, D. McCarthy, S. J. McGowan, M. F. McMullin, K. A. Miller, L. Murray, A. H. Németh, M. A. Nesbit, D. Nutt, E. Ormondroyd, A. B. Oturai, A. Pagnamenta, S. Y. Patel, M. Percy, N. Petousi, P. Piazza, S. E. Piret, G. Polanco-Echeverry, N. Popitsch, F. Powrie, C. Pugh, L. Quek, P. A. Robbins, K. Robson, A. Russo, N. Sahgal, P. A. van Schouwenburg, A. Schuh, E. Silverman, A. Simmons, P. S. Sørensen, E. Sweeney, J. Taylor, R. V. Thakker, I. Tomlinson, A. Trebes, S. R. F. Twigg, H. H. Uhlig, P. Vyas, T. Vyse, S. A. Wall, H. Watkins, M. P. Whyte, L. Witty, B. Wright, C. Yau, D. Buck, S. Humphray, P. J. Ratcliffe, J. I. Bell, A. O. M. Wilkie, D. Bentley, P. Donnelly, and G. McVean. Factors influencing success of clinical genome sequencing across a broad spectrum of disorders. *Nat Genet*, 47(7):717–726, Jul 2015.

[18] K. L. Tucker. Methylated cytosine and the brain: a new base for neuroscience. *Neuron*, 30(3):649–652, Jun 2001.

[19] J. M. Zook, B. Chapman, J. Wang, D. Mittelman, O. Hofmann, W. Hide, and M. Salit. Integrating human sequence data sets provides a resource of benchmark snp and indel genotype calls. *Nat Biotechnol*, 32(3):246–251, Mar 2014.
